# Multi-layered roles of BBX proteins in plant growth and development

**DOI:** 10.1007/s44154-022-00080-z

**Published:** 2023-01-06

**Authors:** Jing Cao, Jiale Yuan, Yingli Zhang, Chen Chen, Beihong Zhang, Xianming Shi, Rui Niu, Fang Lin

**Affiliations:** Ministry of Education Key Laboratory of Cell Activities and Stress Adaptations, School of Life Sciences, Lanzhou University, Lanzhou, 730000 China

**Keywords:** Light, BBX, Photomorphogenesis, Phytohormone

## Abstract

Light and phytohormone are external and internal cues that regulate plant growth and development throughout their life cycle. BBXs (B-box domain proteins) are a group of zinc finger proteins that not only directly govern the transcription of target genes but also associate with other factors to create a meticulous regulatory network to precisely regulate numerous aspects of growth and developmental processes in plants. Recent studies demonstrate that BBXs play pivotal roles in light-controlled plant growth and development. Besides, BBXs have been documented to regulate phytohormone-mediated physiological procedures. In this review, we summarize and highlight the multi-faced role of BBXs, with a focus in photomorphogenesis, photoperiodic flowering, shade avoidance, abiotic stress, and phytohormone-mediated growth and development in plant.

## Introduction

Light regulates plant growth and development during all phases of its life cycle including seed germination, photomorphogenesis, shade avoidance, flowering, and circadian rhythm and so on (Jiao et al. 2007; Kami et al. 2010; Sarmiento 2013). Upon seed germination, plants develop short hypocotyls, opened and green cotyledons in the light which is known as photomorphogenesis or de-etiolation. Whereas seedlings undergo skotomorphogenesis or etiolation with long hypocotyls, apical hooks, and closed and etiolated cotyledons in darkness (Jiao et al. 2007; Cheng et al. 2021). Photomorphogenesis is one of the most studied phenomena for plants to dwell life in presence of light. A comprehensive signaling network has been evolved in plant to adapt the dynamic light environment (Lin et al. 2021). Specific photoreceptors perceive different color of light to initiate signaling cascade. There are several groups of photoreceptors. Red/far-red light are perceived by phytochromes including phytochrome A-E (phyA-E) in Arabidopsis. CRYPTOCHROMES (CRYs), phototropins, and the ZEITLUPE/FLAVIN-BINDING KELCH REPEAT F-BOX 1/LOV KELCH PROTEIN 2 (ZTL/FKF1/LKP2) family members sense UV-A/blue light (350–500 nm). The UV-B light (275–320 nm) is monitored by UV RESISTANCE LOCUS 8 (UVR8). When photoreceptors inactive in the dark, photomorphogenic repressors CONSTITUTIVELY PHOTOMORPHOGENIC 1 (COP1)-SUPPRESSOR OF PHYTOCHROME A (SPA) target light-promoting factors such as ELONGATED HYPOCOTYL 5 (HY5), LONG HYPOCOTYL IN FAR-RED 1 (HFR1) and B-BOX PROTEIN 21 (BBX21) for ubiquitination and degradation in a 26S proteasome dependent manner, contributing to promote skotomorphogenesis. Another group of photomorphogenic repressors are PHYTOCHROME-INTERACTING FACTORS (PIFs) which belongs to a group of basic helix-loop-helix (b-HLH) type transcription factors. PIFs accelerate skotomorphogensis by directly regulating the expression of thousands target genes. In visible light conditions, photo-excited receptors negatively regulate photomorphogenic repressors such as COP1-SPAs and PIFs in the nucleus through multiple dynamic and highly integrated molecular mechanisms, relieving photomorphogenic-promoting factors such as HY5, HFR1, and BBX21 to promote photomorphogenesis (Cheng et al. 2021; Lin et al. 2021; Bian et al. 2022).

BBX proteins are a group of zinc-finger B-box domain contained proteins. Significant progress has been made in understanding the key role of BBX proteins in the light-mediated developmental programs, including seed germination, seedling photomorphogenesis, thermomorphogenesis, floral transition, shade avoidance, petal senescence, and circadian rhythm and so on (Khanna et al. 2009; Gangappa and Botto 2014; Song et al. 2020a; Xu 2020; Yadav et al. 2020). Phytohormones are crucial internal cues monitor plant growth and development. Accumulated studies demonstrate that BBX proteins also play essential role in phytohormone-mediated growth and development procedures such as auxin, gibberellin acid (GA), abscisic acid (ABA), and brassionsteroid (BR) and so on. This review summarizes and highlights the function of BBXs at transcriptional and posttranscriptional level, especially in the process of photomorphogenesis, flowering, shade avoidance, abiotic stress, and phytohormone signaling pathways. In addition, we further provide insights into the potentials of BBXs in crop breeding. To characterize the roles of BBXs in various processes, readers are recommended to other recent research and review articles (Sarmiento 2013; Vaishak et al. 2019; Song et al. 2020a; Xu 2020).

## Domain and structure of BBXs

**Fig. 1:**
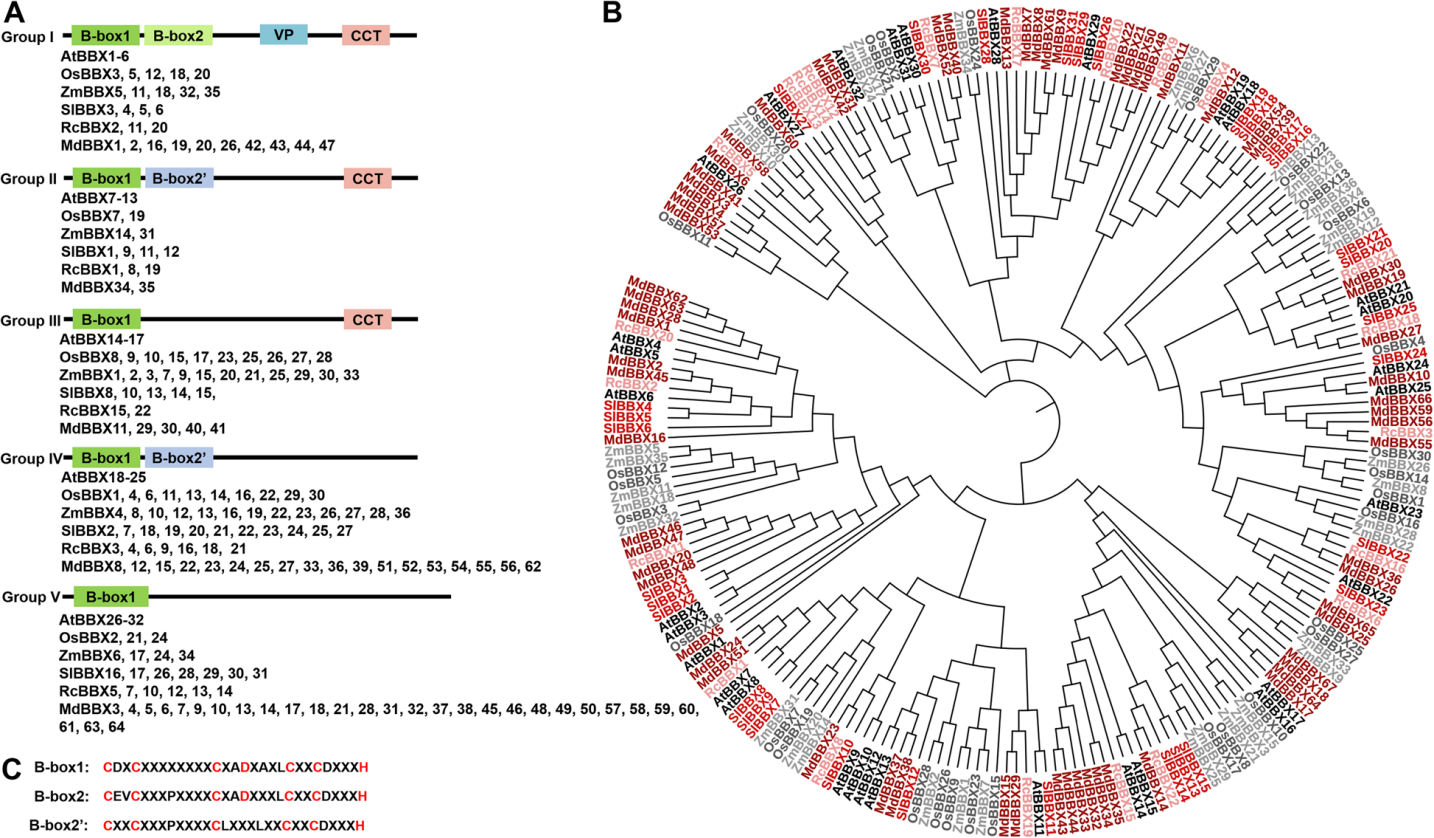
Phylogenetic relationship, protein structure and architecture of the conserved BBX proteins in plants. **A** Structures of the BBX proteins with the composition of the domains in *Arabidopsis thaliana*, rice (*Oryza sativa*), maize (*Zea mays*), tomato (*Solanum lycopersicum*), rose (*Rosa chinesis*) and apple (*Malus domestica Borkh*). **B** Molecular phylogenetic analysis of BBX proteins in *Arabidopsis thaliana*, rice (*Oryza sativa*), maize (*Zea mays*), tomato (*Solanum lycopersicum*), rose (*Rosa chinesis*) and apple (*Malus domestica Borkh*). **C** Consensus sequences of  B-box1, B-box2 and B-box2’ in Arabidopsis, red color indicates the conserved amino acids

BBX proteins have a conserved feature, which is a B-box domain. The B-box domain represents a subgroup of zinc finger motif which is stabilized by the binding of zinc ions and has the property to interact with DNA, RNA, or proteins to precisely regulate growth and developmental process (Khanna et al. 2009). In animals, the B-box domain often associates with RING-finger and coiled-coil domains, forming tripartite motif (TRIM) proteins or RING, B-box, Coiled-coil (RBCC) proteins. The TRIM/RBCC proteins are a class of single protein RING finger E3 ubiquitin ligases, which are involved in several physiological and pathological conditions including Mendelian genetic diseases, cancer development and virial infection (Reymond et al. 2001). By contrast, plant B-box domain either found alone or together with the CCT domain which are referred to as B-box proteins (BBXs). All of the plant BBX proteins are classified into five structure groups depending on the presence of at least one B-box domain and a CCT domain (Fig. 1) (Khanna et al. 2009; Gangappa and Botto 2014). The B-box domain consists of one or two B-box motifs with a length of 40 residues. Based on the consensus sequence and the spacing of the seven or eight Zn-binding residues, the B-box motifs fall into two types, B-box1 and B-box2. Some of the B-box domain not only directly mediate transcriptional regulation, but also interact with other factors to modulate protein activity including binding ability, transcriptional activity, and E3 ubiquitin ligase activity and so on (Gangappa and Botto 2014).

The CCT domain has 42–43 amino acids in length at the C-terminus which was initially found in CONSTANSE, CONSTANSE-like, and TOC1 (TIMING OF CAB EXPRESSION1) proteins. Many reports have indicated that the CCT domain is crucial for its DNA binding ability, transcriptional activity, and nuclear localization. Some BBX proteins contain a CCT domain which play critical role in flowering (Khanna et al. 2009; Song et al. 2020a).

Between the B-box domain and CCT domain, an essential binding sequence motif for protein interaction called the VP pair which also exists in some BBX proteins. The VP pair consists of an array of residues: VP(E/D)φG, in which φ represents a hydrophobic amino acid (Holm and Deng 1999; Holm et al. 2001; Datta et al. 2006). Additionally, seven newly identified motifs (M1 to M7) are also conserved in some BBX proteins. The M1 motif contains the VP pair which belongs to the structure group I. The M6 motif, presenting in the structure group IV, significantly affects the functional determination of AtBBX21 to AtBBX24 (Crocco and Botto 2013; Yadav et al. 2020).

In Arabidopsis, 21 of the 32 BBX proteins contain two B-boxes in tandem, whereas 11 BBX proteins contain one B-box (Khanna et al. 2009; Gangappa and Botto 2014). 17 of the 32 BBX proteins have a CCT domain which are also called CO and CONSTANSE-like (COL) proteins, while others have not. The structure group I contain six members (AtBBX1 to AtBBX6) which consist of two B-boxes (B1 and B2) in tandem and a CCT domain. The structure group II contain seven members (AtBBX7 to AtBBX13) which comprise of B1, B2’, and a CCT domain. The AtBBX members of structure group III (AtBBX14 to AtBBX17) have a B-box domain and a CCT domain. The structure group IV (AtBBX18 to AtBBX25) carry B1 and B2' domain, whereas the structure group V (AtBBX26 to AtBBX32) have only one B-box domain (Khanna et al. 2009).

Recently, accumulated studies indicate that BBX proteins also evolutionally exist among different plant species (Fig. 1) (Talar and Kielbowicz-Matuk 2021). In rice (Oryza sativa), 17 of the 30 BBX proteins contain tandem B-boxes in their N termini (Huang et al. 2012). 36 BBX proteins are identified in maize, while 20 BBX proteins carry two B-box domains (Shalmani et al. 2019, Xu et al. 2022). 21 of 27 BBX proteins contain two B-box motifs in Moso bamboo (Ma et al. 2021). 96 BBX proteins are identified in wheat (Chen et al. 2021). 64 BBX representatives are characterized in apple (Liu et al. 2018), 37 BBX proteins in cotton (*Gossypium hirsutum*) (Feng et al. 2021), 37 BBX proteins in white pear, 30 BBX proteins in potato (Talar et al. 2017), 31 BBX proteins in tomato (Chu et al. 2016; Bu et al. 2021), 56 BBX protein in soybean (Fan et al. 2014), 25 BBX proteins in pear and bananas (Chaurasia et al. 2016; Cao et al. 2017), 24 BBX proteins in grapevine and peanut (Jin et al. 2020; Wei et al. 2020), 22 BBX proteins in rose (*Rosa chinensis*) (Shalmani et al. 2018), 21 BBX proteins in strawberry (*Fragaria vesca*) (Shalmani et al. 2018), 20 BBX proteins in peach and raspberry (Shalmani et al. 2018), and 15 BBX proteins in sweet cherry (Wang et al. 2021).

## BBXs act as transcription factors

The B-box domain has been reported to directly bind to the DNA sequence, indicating it has DNA binding ability, while the CCT domain carries DNA binding activity and transcriptional activity. One BBX protein contain B-box domain and/or CCT domain imply that it has a potential to act as transcription factor. Actually, BBX1/CO (CONSTANS), the first identified B-box protein, belongs to the structure group I which contain two B-box domain and a CCT domain. It has reported that the CCT domain of CO directly associates with the promoter containing a consensus TGTG(N_2-3_)ATG motif (also named as CO-responsive elements, COREs) of *FT (FLOWERING LOCUS T),* activating *FT* expression and flowering (Tiwari et al. 2010). However, CO is also recommended to form a trimer with NUCLEAR FACTOR Y, SUBUNIT B2 (NF-YB2) and NUCLEAR FACTOR Y, SUBUNIT C3 (NF-YC3) to bind the CORE element of *FT* promoter to elevate its expression (Gnesutta et al. 2017). Additionally, mutations in *CO* delay flowering in the long-day conditions, while conditional induction of *CO* causes early flowering under long-day and short-day conditions (Simon et al. 1996). Thus, CO might function as a transcription factor to promote flowering under inductive long day conditions.

BBX11, belongs to the second subgroup, plays a positive role in red light signaling. Overexpression *BBX11* develops short hypocotyl, while mutation in *BBX11* leads to elongated hypocotyl in the red light and long day conditions. It has reported that BBX11 acts as a scaffold to physically interact with phyB and PIF4, enhancing the interaction between phyB and PIF4, which subsequently not only promotes the degradation of PIF4, but also represses the binding ability of PIF4 to its targets and thus inhibits the transcriptional activity of PIF4 (Song et al. 2021). In addition, BBX11 associates with *HY5* promoter to up-regulate its expression, the accumulated HY5 which in turn improves *BBX11* expression through directly binding to its promoter. Such positive feedback loop further promotes seedling photomorphogenesis (Zhao et al. 2020; Job and Datta 2021). Therefore, BBX11 characterizes as a transcription factor in seedling establishment.

BBX19 is a negative regulator of flowering under inductive photoperiod. BBX19 not only physically interacts with CO to repress its transcriptional activity on *FT* expression and flowering, but also associates with COP1 which promotes its E3 ubiquitin ligase activity on ELF3 degradation, leading to repress seedling photomorphogenesis (Wang et al. 2014, 2015). Besides, loss of function and overexpression of *BBX19* exhibit shortened and lengthened circadian period respectively, implicating the negative role of BBX19 in the circadian clock. PSEUDO-RESPONSE REGULATOR9 (PRR9), PRR7, and PRR5, negative components in the circadian clock, interact with BBX19 to facilitate the association of BBX19 with the promoter of morning-phased clock genes such as *CIRCADIAN CLOCK ASSOCIATED 1 (CCA1)*, *LATE ELONGATED HYPOCOTYL 1 (LHY1)*, and *REVEILLE 8 (RVE8),* contributing to enhance the repressive effect on the morning-phased clock genes (Yuan et al. 2021). In addition, BBX19 directly binds to the promoter of *ABI5* to activate its expression, suppressing seed germination (Bai et al. 2019a). Thus, BBX19 acts as a transcription factor to regulate diverse physiological and development response.

BBX21 (also known as SALT TOLERANCE HOMOLOG2, STH2), a photomorphogenesis-promoting factors, belongs to the structure group IV which have two B-box domains in tandem at the N terminus (Datta et al. 2007). Loss of function of *BBX21* displays short hypocotyls, whereas overexpression of *BBX21* leads to dramatic short hypocotyls in various light conditions. It has been reported that BBX21 not only directly binds to the promoter of *HY5*, *BBX22*, and *GIBBERELLIN 2-β-DIOXYGENASE 1 (GA2ox1)* as well as its own promoter to activate their transcription, but also interacts with HY5 to enhance its activity, leading to promote photomorphogenesis (Xu et al. 2016; Bursch et al. 2020). The second B-box domain in BBX21 is essential for its ability to bind to *HY5* promoter (Xu et al. 2018). Thereby, BBX21 is identified as a transcription factor to precisely regulate plant growth and development.

## BBXs act as transcriptional regulators

Some BBX proteins do not have the ability to directly bind to the promoter of target genes, however they could regulate the transcriptional activity of their partners. Some other BBX proteins have the capacity to bind to the promoter of target genes, but don’t have the transcriptional activity. Therefore, most of the BBX proteins act as transcriptional regulators.

BBX4, a positive regulator of red light signaling, associates with PIF3 to repress PIF3 transcriptional activation activity and PIF3-controlled gene expression (Heng et al. 2019a). In addition, BBX4 directly binds to the promoter of *FT* in the presence of BBX32 to repress *FT* expression and flowering (Tripathi et al. 2017).

BBX18 and BBX23 belong to the structure group IV, which contains two tandem B-box domain. It has reported that BBX18 and BBX23 interact with ELF3 to positively regulate thermomorphogenesis which dependent on COP1 and PIF4 (Ding et al. 2018). In addition, BBX18 interacts with PRR5 to alleviate the PRR5-mediated suppression of PIF4 activity, concomitantly enhancing the thermoresponsive hypocotyl growth in a ELF3 independent pathway (Hwang et al. 2021). BBX20, which is transcriptionally repressed by BZR1, promotes photomorphogenesis and represses brassinosteriod signaling pathway (Fan et al. 2012). It has reported that BBX20 interacts with HY5 to enhance its transcriptional capacity, while BBX20 could not activate target gene expression in the absence of HY5 (Fan et al. 2012). In addition, BBX21 and BBX22 work redundantly with BBX20 to interact with HY5 and increase its transcriptional activity to promote photomorphogenesis (Bursch et al. 2020). Besides, BBX23 also physically interacts with HY5 to synergistically regulate the expression of light responsive genes (Zhang et al. 2017). Thus, BBX20-23 and HY5 work largely interdependently to regulate the expression of their targets in photomorphogenic growth in higher plants. BBX24 and BBX25 negatively regulate seedling photomorphogenesis, which are the last two B-Box proteins of structure group IV. BBX24 and BBX25 have been reported to physically interact with HY5 and form heterodimers, interfering HY5 transcriptional activity and repressing photomorphogenesis (Gangappa et al. 2013b, 2013a). In addition, BBX24 interacts with DELLA and mitigates DELLA-mediated suppression of PIF4 activity to promote shade avoidance (Crocco et al. 2015).

BBX28 and BBX29 contain one B-box domain and belong to the subfamily V. It has revealed that BBX28 and BBX29 physically interact with HY5 to inhibit its ability to bind to target sites and regulate gene expression, thereby repressing photomorphogenesis (Lin et al. 2018; Song et al. 2020b). In addition, BBX28 and BBX29 interact with BR ENHANCED EXPRESSION1/2/3 (BEE1/2/3) to enhance their binding ability to their targets and improve the transcriptional activation activity of BEE1/2/3 (Cao et al. 2022). Moreover, BBX28 interferes with the binding of CO to the promoters of *FT* through physical interactions, repressing *FT* expression and flowering under long day condition (Liu et al. 2020). *BBX30* and *BBX31* are transcriptional repressed by HY5, while BBX28 and BBX29 physically interact with HY5 to block its binding ability to the promoters of *BBX30* and *BBX31,* resulting in promoting the expression of these genes (Heng et al. 2019b; Song et al. 2020b). In addition, BBX30 and BBX31 associate with the promoter of *BBX28* and *BBX29*, which in turn increases their transcripts level and forms a positive feedback loop to fine-tune photomorphogenic development (Song et al. 2020b). BBX32, the last member of subgroup V, interacts with BBX21 and suppresses its transcriptional activity in HY5 dependent and independent pathways (Holtan et al. 2011). Besides, BBX32 interacts with BZR1 and PIF3 to promote BR-mediated cotyledon closure, suggesting BBX32 appears to be a co-regulator of BBX21, BRASSINAZOLE-RESISTANT 1 (BZR1), and PIF3 to control target gene expression during seedling establishment (Ravindran et al. 2021). Therefore, BBX4, BBX18, BBX20, BBX22, BBX23, BBX24, BBX25, BBX28, BBX29, BBX30, BBX31, and BBX32 function as transcriptional regulators in diverse growth and developmental processes.

## Functions of BBXs in photomorphogenesis

Photomorphogenesis and skotomorphogenesis are two contrast process in seedling development. In the light, plant undergoes photomorphogenesis with short hypocotyl, opened and expanded cotyledon, and green chloroplast for photoautotrophic growth. In the dark, plant exhibits skotomorphogenesis with elongated hypocotyl, closed and etiolated cotyledon, and apical hook. When photoreceptors are inactive in the dark, photomorphogenic repressors COP1-SPA complex and PIFs are active in the nucleus to promote skotomorphogenesis by targeting photomorphogenesis-promoting transcription factors such as HY5 for degradation and controlling the expression of genes related to cell elongation and expansion, respectively (Lau and Deng 2012; Xu et al. 2015; Cheng et al. 2021). Upon visible light absorption, active photoreceptors repress the activity and accumulation of COP1-SPA complex and PIFs through multiple mechanisms, relaying light signaling to promote photomorphogenesis. Extensive studies have reported that BBX proteins act as key factors in the COP1-HY5 regulatory hub to regulate light-mediated seedling development.

**Fig. 2:**
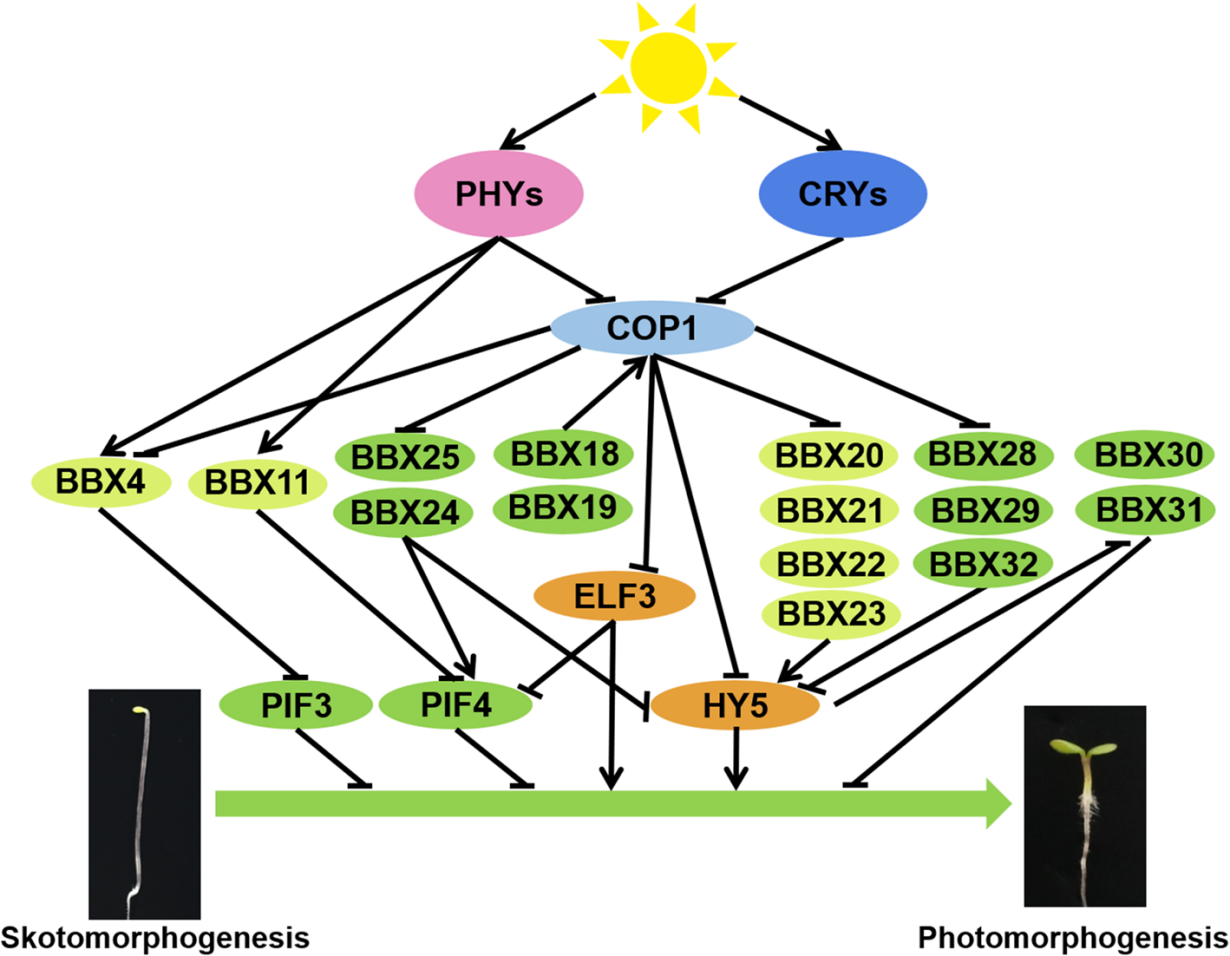
BBX proteins in plant photomorphogenesis. PhyB interacts with BBX4 to promote its accumulation, the accumulated BBX4 interacts with PIF3 to repress its transcriptional activation activity. BBX11 interacts with phyB which enhances the interaction between phyB and PIF4, leading to improve the degradation of PIF4 and promote photomorphogenesis in the prolonged red light. In addition, BBX11 interacts with PIF4 to repress the binding ability of PIF4 to its targets which further contributes to enhance plant photomorphogenesis. BBX18 and BBX19 associate with COP1 to elevate its E3 ubiquitin ligase activity towards ELF3. COP1 targets BBX20, BBX21, BBX22, BBX23, BBX24, BBX25, BBX28 and BBX29 for degradation. BBX20, BBX21, BBX22 and BBX23 interact with HY5 to promote its transcriptional activation activity to facilitate plant photomorphogenesis, whereas BBX24, BBX25, BBX28, BBX29, and BBX32 interact with HY5 to form heterodimers and repress its transcriptional activity. HY5 directly binds to the promoter of *BBX30* and *BBX31* to repress their expression, while BBX30 and BBX31 inhibit plant photomorphogenesis via regulating genes-related to hormone and cell elongation

BBX4, BBX11, BBX20, BBX21, BBX22, and BBX23 positively regulate seedling photomorphogenesis, while BBX18, BBX19, BBX24, BBX25, BBX28, BBX29, BBX30, BBX31 and BBX32 repress photomorphogenesis in response to a wide range of light signals (Fig. 2). BBX4 specifically mediates red light-controlled seedling development, as the single mutant displays elongated hypocotyls in the red light and overexpression of *BBX4* exhibits significantly shortened hypocotyls in various light conditions (Heng et al. 2019a). phyB is the primary photoreceptor for red light which photoconvert to the activated far-red light-absorbing form (Pfr), whereas far-red light irradiation reverses the activated phytochromes to red light-absorbing form (Pr). Phytochromes are synthesized in the cytosol with the Pr form. Upon red light irradiation, biologically active Pfr form of phyB translocate into the nucleus to form nuclear bodies with COP1, SPAs, PIFs and BBX4. These molecular events trigger the repression of E3 ubiquitin ligase activity of COP1-SPA complex, the phosphorylation, ubiquitination and degradation of PIFs, and the accumulation of BBX4. In addition, BBX4 interacts with the remaining pool of PIF3 to form heterodimers and repress its transcriptional activity, reinforcing the phyB-mediated inactivation of PIF3 and thereby promoting photomorphogenesis (Heng et al. 2019a). BBX20, BBX21, BBX22 and BBX23 interact with HY5 to strength its transcriptional activity, eventually promote photomorphogenesis (Zhang et al., 2017a; Bursch et al. 2020). Moreover, BBX20, BBX21, and BBX22 upregulate the transcript level of *HY5* to further accelerate photomorphogenic development (Xu et al. 2016; Bursch et al. 2020).

BBX18, also named double B-box 1a (DBB1a), is the first member of B-box family that only contain two B-box motifs. BBX18 co-suppressed and over-expressed transgenic lines exhibit shortened and elongated hypocotyls respectively in the blue light (Wang et al. 2011). Thus, BBX18 acts as a negative regulator of blue light-controlled photomorphogenesis. BBX19 also promotes hypocotyl growth, as the RNA interference line reduces hypocotyl length while the constitutive expression line increases hypocotyl elongation. BBX19 acts as an adaptor to strength COP1-mediated degradation of ELF3 through physically interacts with COP1 and ELF3, alleviating the repression of *PIF4/5* by evening complex and thus repressing photomorphogenic development (Wang et al. 2015).

BBX24 and BBX25 additively promote hypocotyl elongation. In different light conditions, *bbx24-1 bbx25-1* double mutant seedlings are significantly shorter than that of each of single mutants which are shorter than wild type, whereas *BBX25*-overexpressing lines exhibit longer hypocotyls. Biochemical data indicate that BBX24 and BBX25 interact with HY5 to form heterodimers to repress HY5 transcriptional activity. Thus, BBX24 and BBX25 work redundantly to repress seedling photomorphogenesis (Gangappa et al. 2013b). In addition, BBX24 not only represses the transcriptional activity of HY5 to negatively regulate UV-B-mediated photomorphogenic response, but also interacts with DELLA to derepress PIF4 activity and eventually promote shade avoidance (Jiang et al. 2012; Crocco et al. 2015).

Mutations of *BBX28, BBX29, BBX30* and *BBX31* (quadruple mutants) develop short hypocotyls which is shorter than the double and single mutants in various light conditions, while overexpression *BBX28*, *BBX29*, *BBX30* and *BBX31* markedly promote hypocotyl elongation, suggesting BBX28, BBX29, BBX30 and BBX31 additively repress seedling photomorphogenesis (Song et al. 2020b). BBX28 and BBX29 associate with HY5 to form heterodimeric complex which interfere with the binding ability of HY5 to its targets such as *BBX30* and *BBX31*, subsequently increasing the transcripts level of *BBX30* and *BBX31,* whose proteins bind the promoter of *BBX28* and *BBX29* to upregulate the expression of these genes (Lin et al. 2018; Song et al. 2020b)*.* This positive feedback loop further assists to repress photomorphogenesis to fine-tune seedling development in response to various light conditions.

Seedlings overexpressing *BBX32* display elongated hypocotyl and hyposensitivity to various light conditions, whereas *bbx32* mutants exhibit short hypocotyls in low fluence light conditions. BBX32 acts as transcriptional regulators to interact with BBX21 and repress its transcriptional activity in a HY5 dependent or independent pathway, eventually leading to repress seedlings photomorphogenesis (Holtan et al. 2011).

## Functions of BBXs in flowering

**Fig. 3:**
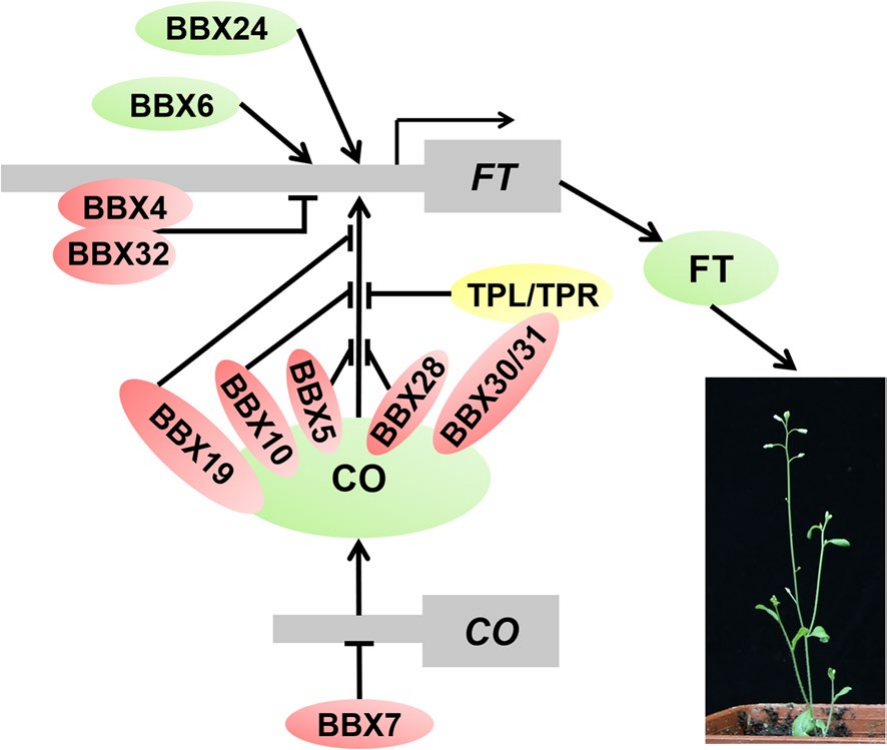
BBX proteins-mediated flowering. CO, BBX6, BBX24 promote flowering via activate *FT* expression, whereas BBX7 inhibits flowering through repressing the expression of *CO*. BBX5, BBX10, BBX19, and BBX28 interact with CO to repress its transcriptional activity, resulting in delaying flowering. BBX30 and BBX31 act as scaffold to bridge CO and TPL/TPR, leading to block the transcriptional activation activity of CO towards *FT*. BBX proteins in red circle such as BBX4, BBX5, BBX7, BBX10, BBX19, BBX28, BBX30, BBX31 and BBX32 are flowering repressors, whereas BBX proteins in green circle such as CO, BBX6 and BBX24 represent flowering inducers

Extensive studies have shown that multiple B-box proteins play critical role in floral regulation. The first characterized and best identified member of B-box family in Arabidopsis was CO/BBX1 which is a crucial activator of flowering under inductive long days. CO acts as a transcription factor to directly bind the promoter of *FLOWERING LOCUS T (FT)* via its CCT domain, eventually leading to activate *FT* expression and initiate flowering (Tiwari et al. 2010). The CO-FT module play essential role in photoperiodic flowering and is highly conserved across various plant species such as rice, barley, maize, tomato, and sunflowers. Besides, other members of BBX family such as BBX4/COL3, BBX5/COL4, BBX6/COL5, BBX7/COL9, BBX10/COL12, BBX19, BBX24, BBX28, BBX30/miP1a, BBX31/miP1b, and BBX32 have been reported to mediate flowering through distinct mechanisms, either positively or negatively. CO, BBX6, and BBX24 accelerate flowering, while BBX4, BBX5, BBX7, BBX10, BBX19, BBX28, BBX30, BBX31, and BBX32 delay flowering (Fig. 3).

BBX6, which is transcriptionally regulated by GIGANTEA (GI), functions as a flowering activator. Overexpression of *BBX6* causes early flowering under short days, while loss of function of *BBX6* do not show altered flowering which may be due to inappropriately expression of *BBX6* or the redundantly with other B-box proteins (Hassidim et al. 2009). BBX24, also named STO (SALT TOLERANCE), functions as a positive regulator of flowering in photoperiod pathway. Overexpression of *BBX24* accelerates flowering under both long-day and short-day conditions, while loss of function of *BBX24* delays flowering only in short-day conditions. BBX24 not only represses *FLOWERING LOCUS C (FLC)* expression, but also promote *FT* and *SUPPRESSOR OF OVEREXPRESSION OF CO 1 (SOC1)* expression in a CO-independent manner, consequently leading to promote flowering (Li et al. 2014).

The mutant of *BBX4* exhibits early flowering, while overexpression of *BBX4* shows a late flowering phenotype under long day conditions like what is observed for *BBX32-OX*. However, the artificial microRNA line of *BBX32* also flowered late in both long-day and short-day conditions, which could be due to the redundancy and/or feedback regulation of B-box family proteins. Biochemical data demonstrate that BBX4 binds to the promoter region of *FT* in the presence of BBX32 to repress *FT* expression, delaying flowering under long day conditions (Tripathi et al. 2017). The molecular lesion in *BBX5/COL4* results in increasing expression of *FT* and *AP1* which accelerate flowering under long day conditions, whereas *BBX5* overexpression lines show delayed flowering. BBX5 colocalizes with CO in nuclear speckles which may interfere the process of CO-activated *FT* expression. Thus, BBX5 is a flowering repressor (Steinbach 2019). BBX7/COL9 is another floral repressor in photoperiod pathway. Overexpression of *BBX7* renders late flowering, while loss of function of *BBX7* displays early flowering under long day conditions. BBX7 antagonistically represses the expression of *CO* and concomitantly reduces the expression of *FT* and *SOC1* to delay flowering (Cheng and Wang 2005). Thus, BBX7 transcriptionally represses *CO, FT* and *SOC1* expression to suppress floral transition. Thereby, BBX4, BBX5, BBX7 inhibit flowering through attenuating *CO* and/or *FT* expression.

Overexpression of *BBX10/COL12* delays flowering under long day conditions. BBX10 sequesters CO into non-functional complex to repress its activity toward *FT,* leading to decline *FT* expression and delay flowering. In addition, BBX10 interacts with COP1-SPA1 complex which target BBX10 for degradation in the dark (Ordonez-Herrera et al. 2018). BBX19 and BBX28 are also floral repressors. Constitutive expression of *BBX19* and *BBX28* cause late flowering in inductive photoperiods, whereas loss of function of these genes accelerate flowering. BBX19 interacts with CO to form heterodimers to inhibit the activity of CO toward *FT,* resulting in the low *FT* expression and late flowering (Wang et al. 2014). BBX28 decreases the binding ability of CO to *FT* locus via physical interaction with CO, leading to reduce the transcript of *FT* and negatively regulate flowering (Liu et al. 2020). As short, single domain proteins, BBX30 and BBX31 are also named MicroProteins1a and MicroProteins1b which are only contain one B-box domain. MicroProteins act as important modulators to avert large, multi-domain proteins from forming functional multimers. BBX30 and BBX31 interact with CO, as well as recruit TPL/TPR co-repressor proteins via additional PFVFL motif to attenuate CO function. Overexpression *BBX30* and *BBX31* render severely delayed flowering, while *bbx30 bbx31* double mutants show early flowering under long day conditions (Graeff et al. 2016; Heng et al. 2019b). Thus, BBX30 and BBX31 act as adaptors to bridge TPL/TPR co-repressor proteins and CO transcription factors to repress CO activity, leading to attenuate *FT* expression and delay the transition to flowering.

CO-FT module is a predominant regulatory hub of floral initiation, numerous BBXs regulate the transcript level or transcriptional activation activity of CO via diverse regulatory mechanisms to modulate *FT* expression and flowering process. Moreover, a group of BBXs control *FT* expression and reproductive development independent of CO. Plants exert the BBXs-mediated regulatory networks to fine-tune photoperiodic flowering in response to the changeable environment.

BBX family are highly conserved in green plant. For example, there are 30 BBX members in rice, and most of them are also reported to play vital role in floral transition. Rice HEADING DATE 1 (HD1/OsBBX18), which is an orthologue of CO in Arabidopsis, promotes *Hd3a (OsFT)* expression to activate flowering in short days while suppresses *Hd3a* expression to inhibit flowering in long day conditions (Yano et al. 2000). Dissimilar to HD1, a group of B-box containing proteins are proved to repress flowering in rice. Overexpression of *OsCO3* in rice displays delayed heading date in short day conditions via repressing *FT*-like genes (Kim et al. 2008). Loss of function of *OsCOL4* (*OsBBX5*) or *OsCOL9* (*OsBBX7*) flower early, while gain of function of *OsCOL4* or *OsCOL9* flower late under short or long days. Both OsCOL4 and OsCOL9 negatively regulate *EARLY HEADING DATE 1 (Ehd1)*, *Hd3a*, and *RICE FLOWERING LOCUS T1* (*RFT1)* expression to suppress flowering (Lee et al. 2010; Liu et al. 2016a, b). Overexpression of *OsBBX14* causes late flowering regardless of day length through declining *Hd3a* and *RFT1* expression (Bai et al. 2016). Overexpression of *OsCOL10* and *OsCOL13* delay flowering under long-day and short-day conditions via repressing *Ehd1, Hd3a*, and *RFT1* expression. OsCOL10 (OsBBX10) is positively regulated by long day (LD)-specific flowering suppressor GRAIN NUMBER, PLANT HEIGHT AND HEADING DATE 7 (Ghd7), while OsCOL13 is functionally redundant with OsCOL4 which acts downstream of *OsphyB* and upstream of *Ehd1* to repress flowering (Sheng et al. 2016; Tan et al. 2016, 2017). Overexpression of *OsCOL15 (OsBBX26)* and *OsCOL16 (OsBBX17)* trigger late heading under both long-day and short-day conditions. Both OsCOL15 and OsCOL16 up-regulate the transcripts of *Ghd7* to repress heading via reducing the expression of *Ehd1, Hd3a*, and *RFT1* (Wu et al. 2017, 2018)*.* Thus, OsCO3, OsCOL4, OsCOL9, OsCOL10, OsCOL13, OsCOL15, and OsCOL16 delay flowering under short day and/or long day conditions.

BBX proteins also play essential role in flowering plant. Overexpression of *Chrysanthemum morifolium BBX8 (CmBBX8)* accelerates floral transition, while artificial microRNA-mediated knockdown lines attenuate flowering in summer chrysanthemum. CmBBX8 promotes *CmFTL1* expression to accelerate flowering via directly binding to the promoter of *CmFTL1* (Wang et al. 2020). Heterologous expression of *CmBBX13* in Arabidopsis delays flowering regardless of the day length (Ping et al. 2019). RNA interference-suppressed *CmBBX24* lines flower early than wild type, while ectopic expression of chrysanthemum *CmBBX24* in Arabidopsis delay flowering partially via GA pathway under long day conditions (Yang et al. 2014). Thus, CmBBX13 and CmBBX24 repress but CmBBX8 promotes floral transition in chrysanthemum.

## Functions of BBXs in shade avoidance

**Fig. 4:**
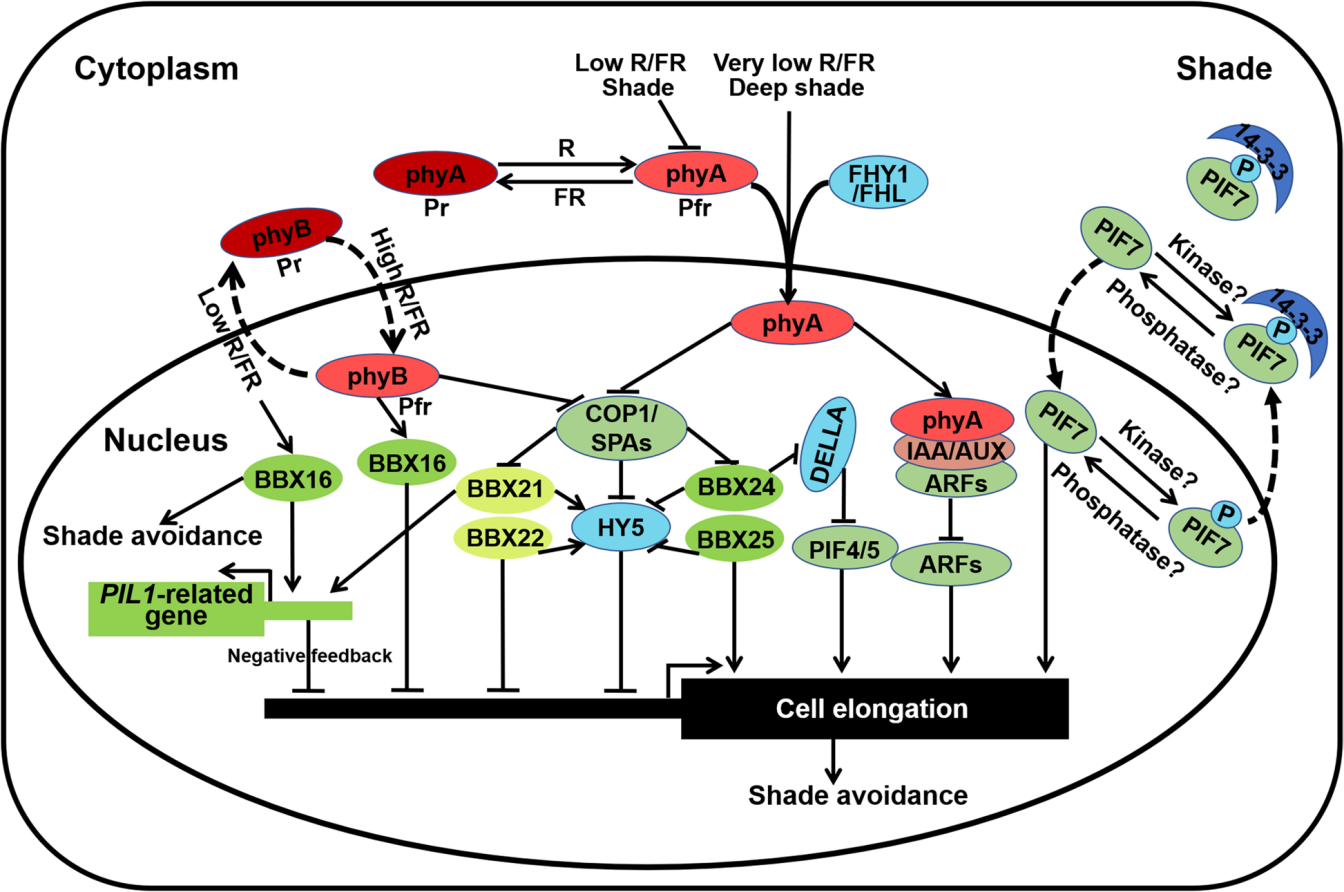
BBX proteins in shade avoidance. When the R/FR ratio is in the range of 0.8–1.0, plants will develop shade avoidance. However, once the ratio of R/FR is reduced to approximately 0.05–0.7, plants will undergo deep shade procedure. Shade condition promotes phyB in the Pr form which is a bio-inactivate state. The Pr form of phyB represses its translocation from the cytoplasm to the nucleus and inhibit the association with photomorphogenic repressors (COP1, SPAs, and PIFs and so on), resulting in promoting HY5, BBX21 and BBX22 degradation, PIFs accumulation, and hypocotyl elongation, thus facilitating shade avoidance. PIF7 is the master regulator to mediate shade-triggered hypocotyl elongation. Shade induces the accumulation of dephosphorylated PIF7 in the nucleus, while white light increases the level of phosphorylated PIF7 which tends to transport into the cytoplasm and interacts with 14–3-3 proteins. Besides, PIF4 and PIF5 contribute to shade-induced hypocotyl elongation by regulating gene expression involved in auxin signal. phyA is the primary photoreceptor in the low ratio of R/FR to mediate shade response. Whereas, deep shade-triggered phyA nuclear accumulation promotes the interaction of phyA and IAA/AUX, leading to repress ARFs activity and inhibit hypocotyl elongation. BBX24 and BBX25 interact with HY5 to repress its activity to promote shade-triggered hypocotyl elongation. Besides, BBX24 associates with DELLA to alleviate the inhibition of PIF4, also releasing hypocotyl growth in the shade. In addition, BBX16 induces hypocotyl elongation in low R:FR, consisting with the improvement of *PIL1* expression

When grown in dense stands, seedlings elongate their hypocotyl in an effort to reach light which is a process termed shade avoidance syndrome (SAS). A reduction in the ratio of red to far-red light and low blue light invoke shade avoidance response which is perceived by phytochromes and cryptochromes. Besides stem elongation, shade avoidance also evokes a suite of adaptive responses including reduced branching, hyponastic leaf orientation, early flowering, and accelerated senescence (Casal 2013; Pierik and de Wit 2014). In the shade, reduced biologically photoreceptors restore COP1 nuclear accumulation, as well as release PIFs and the COP1-SPA complex to promote hypocotyl elongation (Lorrain et al. 2008; Pacin et al. 2013; Leivar and Monte 2014). ARFs-mediated auxin signal also contributes to the shade-induced hypocotyl elongation. However, deep shade-triggered nuclear accumulated PHYA interacts and stabilize with AUX/IAA, resulting in the repression of auxin cascade and inhibition of hypocotyl growth (Yang et al. 2018) (Fig. 4). It is interesting to investigate how deep shade promote the nuclear abundance of PHYA. PIF7 is considered to be a master regulator of shade-induced hypocotyl elongation, which plays essential role in low red: far-red (R:FR) light mediated shade avoidance (Leivar et al. 2008; Li et al. 2012). In contrast to PIF1/3/4/5, PIF7 is relatively light-stable. Far-red light absorbing form of phyB interacts with phosphorylated PIF7, as well as 14–3-3 proteins bind and retain phosphorylated PIF7 in the cytoplasm, thus resulting in the repression of PIF7 target gene expression and inhibition of hypocotyl elongation. However, shade induced the decreasing of active form of phyB and accumulation of de-phosphorylated PIF7 in the nucleus, which lead to shade-triggered hypocotyl elongation (Li et al. 2012). Thereby, the nuclear import of de-phosphorylated PIF7 is triggered by shade response, which is suppressed by 14–3-3 proteins-mediated retention of PIF7 in the cytoplasm (Fig. 3) (Huang et al. 2018). More detailed work on the kinase and phosphatase of PIF7 will add new insights into the PIF7-mediated shade-triggered hypocotyl elongation. Additionally, PIF4 and PIF5 control the expression of multiple phytohormones- and cell elongation-related genes to promote hypocotyl growth, which play redundant role with PIF7 in shade avoidance (Lorrain et al. 2008; Li et al. 2021a).

Accumulated studies indicate that BBX proteins play essential role in shade avoidance, especially the members of group IV which oppositely control shade avoidance-triggered hypocotyl elongation. BBX21 and BBX22 has been reported to negatively regulate long-term canopy shade, while BBX18, BBX24 and BBX25 are positive regulators of shade avoidance (Crocco et al. 2011; Gangappa et al. 2013b). COP1 positively regulates shade avoidance mediated hypocotyl elongation. Mutations of *BBX21* and *BBX22* partially restore the impaired SAS of *cop1* mutant seedlings grown under a low R:FR ratio. Besides, BBX21 positively regulates the expression of *PAR1*, *HFR1*, *PIL1* and *ATHB2* which are early shade avoidance genes. Thus, BBX21 and BBX22 act downstream of COP1 to modulate hypocotyl elongation in a negative feedback loop to avoid exaggerate elongation response under shade condition (Crocco et al. 2011).

BBX24 and BBX25 are redundant in promotion of shade avoidance-induced hypocotyl elongation, which are dependent on COP1. BBX24 and BBX25 interact with HY5 to interfere with its transcriptional activity (Gangappa et al. 2013b). Besides, BBX24 interacts with DELLA proteins to derepress PIF4 activity, releasing PIF4-mediated hypocotyl elongation in shade conditions (Crocco et al. 2015).

BBX16/COL7 promotes shoot branching in high R:FR conditions which is efficiently inhibited by shade conditions. BBX16 triggers hypocotyl elongation in low R:FR, contaminating with inducing *PIL1* expression, whereas BBX16 inhibits shoot growth in moderate or high R:FR to promote photomorphogenesis (Wang et al. 2013b, a). In addition, moderate light increases the expression of *BBX16* which is activated by GLK1 to promote photomorphogenesis, while GUN1 represses *BBX16* transcription through repressing *GLK1* expression to attenuate hook unfolding and cotyledon separation after chloroplast damage (Veciana et al. 2022). Thus, BBX16 acts downstream of GLK1 to repress GUN1-mediated suppression of photomorphogenesis during retrograde signaling activation.

## Functions of BBXs in hormonal signaling

**Fig. 5:**
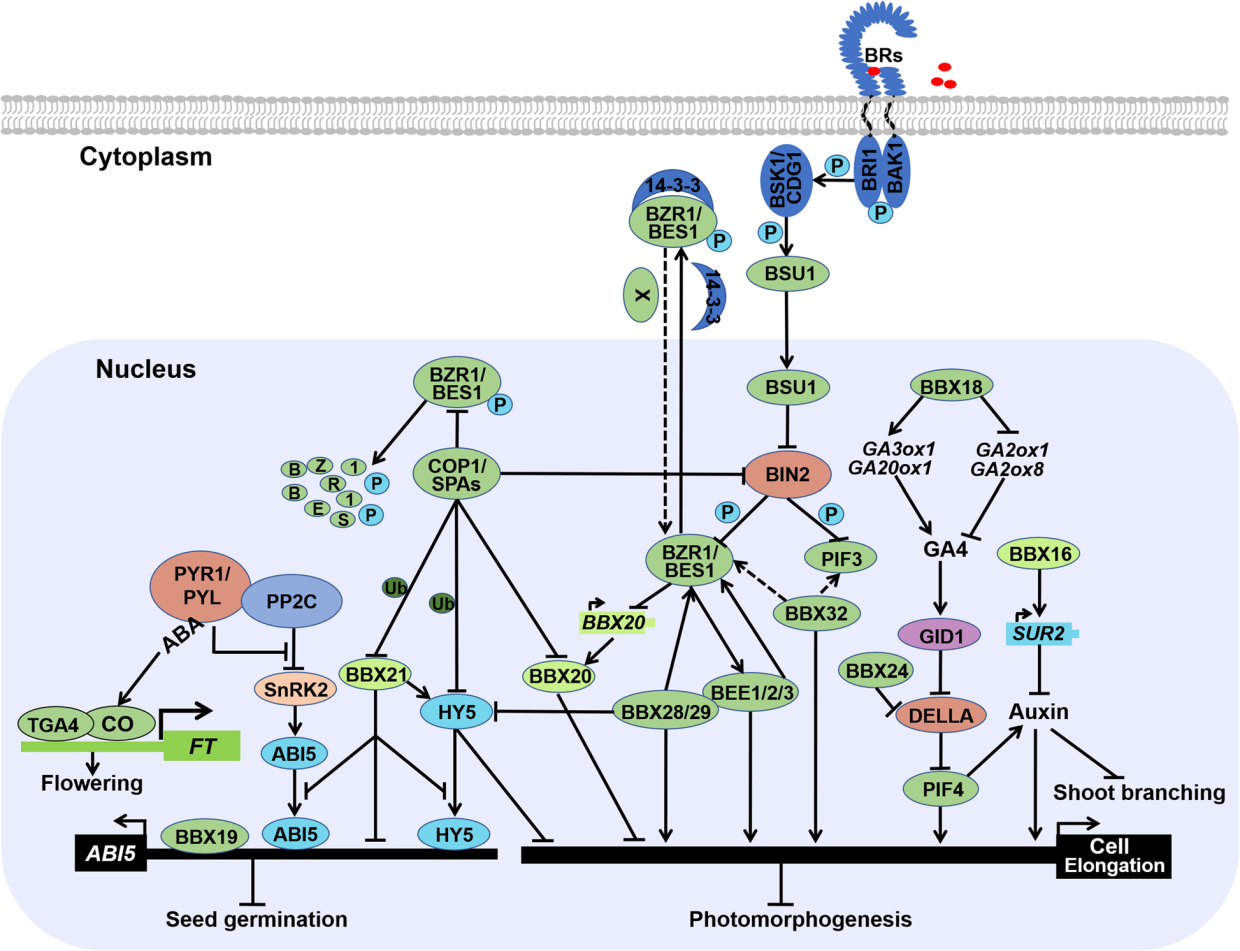
BBX proteins in phytohormone-mediated growth and development. BBX proteins are involved in phytohormone signaling to control plant growth and development. BBX16 augments *SUR2* expression to reduce auxin level. BBX18 increases GA level via elevating GA catabolic related genes and suppressing GA metabolic related genes to promote hypocotyl elongation. BBX24 interacts with DELLA to alleviate DELLA-mediated inhibition of PIF4. BBX32 interacts with BZR1 to control BR-induced cotyledon closure. BZR1 represses the gene expression of *BBX20*. BBX21 associates with HY5 and ABI5 to repress the expression of *ABI5* to promote seed germination. BBX19 directly binds to the promoter of *ABI5* to inhibit seed germination. In addition, ABA induces flowering possibly via improving the transcriptional activity of CO

Light is a key external stimulus to influence plant growth and development, while phytohormones are critical internal cue to regulate a web of physiological responses in plants. Multiple evidences have revealed that BBX proteins play integrated role in phytohormones signaling pathway-mediated cellular and developmental process (Fig. 5). Light promotes the protein accumulation of BBX16 in a phyB-dependent manner. BBX16 activates the transcription of *SUPERROOT 2 (SUR2)*, which is a suppressor of auxin biosynthesis, contributing to reduce auxin level and promote shoot branching in high R:FR light conditions (Zhang et al. 2014). Thus, BBX16 integrates light and auxin to regulate plant shoot branching in response to high light conditions.

BBX18 contains double B-box (DBB) in the N terminus which belongs to subgroup IV. Blue light induces the expression of *BBX18*, which is in a CRY1 and CRY2 dependent manner. Overexpression *BBX18* leads to long hypocotyls in the blue light, while co-suppression of *BBX18* exhibits short hypocotyls. BBX18 not only positively regulates the expression of *GA3β-hydroxygenase1 (GA3ox1)* and *GA20-oxidase1 (GA20ox1)* which are GA biosynthesis genes, but also negatively regulates the expression of GA metabolic genes such as *GA2-oxidase1 (GA2ox1)* and *GA2ox8* under blue light (Wang et al. 2011). Thus, BBX18 suppresses blue light-inhibition of hypocotyl elongation through increasing bioactive GA levels in plant. Mutations in *BBX24* develop short hypocotyls, while overexpression of *BBX24* displays longer hypocotyls in shade conditions. Gibberellin treatment rescues the defects of shade-response in *bbx24* by degradation DELLA proteins which are negative regulators of GA. BBX24 interacts with DELLA proteins to derepress the activity of PIF4 to its targets (Crocco et al. 2015). Thus, BBX24 promotes shade avoidance partially by alleviating DELLA-mediated repression of PIF4 activity. Thereby, BBX24 is a positive regulator of GA signaling by interacting and inhibiting DELLA activity, whether BBX24 affects the interaction between DELLA and GID1 needs further investigation.

In response to drought stress, plant accelerates flowering time and set seeds before more severely drought conditions coming. In this process, plant exerts Abscisic acid (ABA) to promote floral transition under LD conditions partially through inducing the transcriptional activation of CO and/or its downstream targets gene expression such as *FT* (Riboni et al. 2016). Thus, CO coordinates ABA, flowering, and circadian rhythm signaling to balance plant survival and seed set. TGACG MOTIF-BINDING FACTOR 4 (TGA4), named as octopine synthase (ocs)-element-binding factor 4 (OBF4), directly binds to the promoter of *FT* to regulate its expression might through formation the complex with CO. TGA4 also associates with NPR1 in SALICYLIC ACID (SA)-induced leaves, suggesting CO might orchestrate flowering and SA-mediated defense responses to optimize plant growth and development (Song et al. 2008). BBX19, the closet homologous of BBX18, negatively regulate seed germination by promoting ABA signaling. Overexpression of *BBX19* exhibits ABA hypersensitive, while *BBX19* RNA interference lines are insensitive to ABA-mediated suppression of seed germination. Further biochemical data indicate that BBX19 directly binds to the GT1 motif (GGTTAA) of *ABI5* promoter to accelerate its expression (Bai et al. 2019a). Thus, BBX19 coordinates ABA signaling pathway to fine-tune seed germination. Loss of function of *BBX21* are more sensitive to ABA-mediated inhibition of seed germination and stomatal opening, implying BBX21 negatively regulate ABA signaling. Further analysis indicates that BBX21 interacts with HY5 and ABI5 to disrupt the binding ability of HY5 and ABI5 to the promoter of *ABI5*, leading to repress *ABI5* expression and ABA signaling. Thereby, BBX21 integrates light and ABA to work in concert to regulate seed germination (Xu et al. 2014). In addition, BBX21 negatively regulates shade avoidance partially through downregulating the expression of genes associated with auxin, brassinosteroid and ethylene signaling pathways (Crocco et al. 2011). It has been reported that BBX21 interacts with HY5 to promote its transcriptional activity to augment plant photomorphogenesis, however BBX21 associates with HY5 to block its binding capacity towards *ABI5* and suppress ABA-mediated inhibition of seed germination. Thus, BBX21 improves HY5 activity in photomorphogenesis but repress HY5 binding ability in ABA signaling. The contrast role of BBX21 towards HY5 in diverse developmental process awaits further analysis.

Overexpression of *BBX20* develops short hypocotyls, while co-suppression of *BBX20* exhibits slightly but significantly longer hypocotyls compared to wild type in blue and red light. COP1 interacts with BBX20 to induce its degradation in the dark, whereas light triggers the inhibition of COP1 to promote the protein accumulation of BBX20 which leads to promote plant photomorphogenesis. Thus, BBX20 undergoes COP1-mediated degradation in the dark, while light promotes the accumulation of BBX20 through inactivating COP1 to positively regulate plant photomorphogenesis. BBX20, also known as *bzr1–1D* suppressor1-Dominant (BZS1), which is transcriptionally repressed by BZR1. Gain of function of *BZS1* partially suppresses the BR hypersensitive mutants *bzr1-1D* by repressing many BR-activated genes, while knockdown of *BZS1* partially suppress the short hypocotyl of BR-deficient or insensitive mutants (Fan et al. 2012). Thus, BBX20 not only positively regulates light signaling, but also negatively regulates brassinosteroid (BR) signaling. Therefore, BBX20 acts as a node to coordinate light and brassinosteroid signaling to fine-tune plant growth and development. BBX28 and BBX29 associate with HY5 to interfere with the binding ability of HY5 to its targets, leading to repress plant photomorphogenesis. BR induces the protein accumulation of BBX28 and BBX29 which partially dependent on BRI1 and BIN2. Mutations in *BBX28* and *BBX29* partially suppress the constitutively skotomorphogenic phenotype of *bzr1-1D* under treatment of brassinazole (Brz) in the dark, while overexpression of *BBX28* and *BBX29* rescue the short hypocotyl of *bri1* and *bin2-1* partly in the dark and light conditions. Besides, BBX28 and BBX29 interact with BEE1/2/3 which are a group of bHLH type transcription factors to enhance the binding ability of BEE1/2/3 to their targets, leading to promote BR signaling and hypocotyl elongation (Cao et al. 2022). Thus, BBX28 and BBX29 act as a link between light and brassinosteroid signaling to harmonize plant growth and development. BBX32 is highly expressed in the cotyledons, which negatively regulates light signaling but positively promotes BR signaling to repress cotyledon opening during de-etiolation. *bbx32* and *BBX32* overexpression seedlings develop enhanced and reduced cotyledon opening in the light or under treatment of Brz in the dark, respectively. Biochemical data demonstrate that BBX32 interacts with PIF3 and BZR1 to trigger BR-mediated cotyledon closure by regulation the expression of the targets of PIF3 and BZR1 (Ravindran et al. 2021). Thus, these data suggest that BBX32 acts as a hub to converge light and BR signaling to modulate cotyledon closure during the transition from dark to light.

BBX proteins in economic plants also play critical roles in phytohormone-mediated plant growth and development. *CmBBX19*-overexpressing and *CmBBX19*-suppressed lines in chrysanthemum exhibit reduced and enhanced drought stress tolerance respectively. Drought stress and ABA treatment reduce the expression of *CmBBX19.* Besides, CmBBX19 interacts with CmABSCISIC ACID RESPONSIVE ELEMENTS-BINDING FACTOR 3 (CmABF3) which is a master ABA signaling component to suppress its transcriptional activity, leading to inhibit ABA-mediated drought tolerance. Thus, CmBBX19 controls drought stress in an ABA-dependent pathway (Xu et al. 2020). Heterologous overexpression of *CmBBX22* in Arabidopsis improves drought tolerance possibly through up-regulating the expression of ABA cascade related genes such as *ABI3*, *ABI5* and *HY5.* In addition, CmBBX22 also reduces chlorophyll degradation- and leaf senescence-related genes such as *ABF4*, *SAG29* and *NYE1/2* to delay Arabidopsis senescence (Liu et al. 2019a, b). Transgenic plants with suppressed expression of *CmBBX24* (*CmBBX24-RNAi*) in *Chrysanthemum morifolium* hasten time to flower and reduce tolerance to freezing and drought stress. CmBBX24 not only negatively regulates GA biosynthesis and photoperiod pathway related genes such as *GA20OX*, *GA3OX*, and *CmFTL3* respectively, but also positively regulate genes related to compatible solutes and carbohydrate metabolism which are associated with abiotic stress. Under long day conditions, endogenous active GA levels such as GA1 and GA4 in young leaves were increased in *CmBBX24-RNAi* lines but decreased in *CmBBX24* overexpression *(CmBBX24-OX)* plants (Yang et al. 2014). Thus, CmBBX24 delays flowering time and enhances abiotic stress tolerance in chrysanthemum, at least in part by decreasing GA concentrations.

*OsBBX11*, *OsBBX24* and *OsBBX27* are down-regulated, whereas *OsBBX6* is up-regulated under treatment of NAA (a member of the auxin family), GA3 (a gibberellin) and KT (a cytokinin). NAA and KT treatments induce *OsBBX8* expression, while GA3 treatment suppresses the expression of *OsBBX21*. *OsBBX18* and *OsBBX28* are specifically induced with NAA treatment (Huang et al. 2012). Thus, OsBBX proteins also response to phytohormones to modulate rice growth and development.

## Functions of BBXs in abiotic stress response

**Fig. 6:**
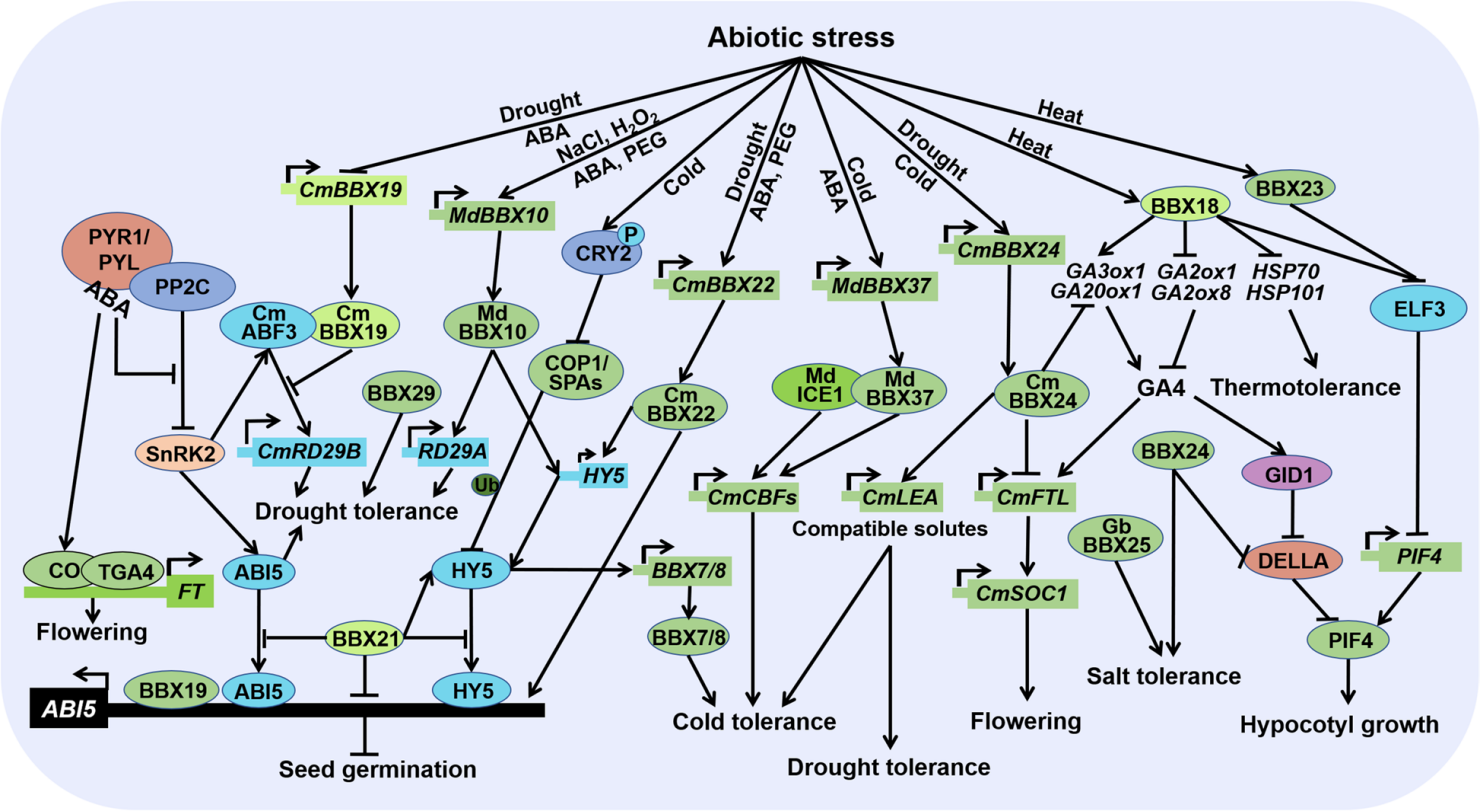
BBX proteins in abiotic stress response. BBX proteins play critical roles in abiotic stress. Drought and ABA repress the expression of *CmBBX19*, whose protein interacts with CmABF3 to inhibits its transcriptional activity, contributing to suppress drought tolerance. The expression of *MdBBX10* is significantly induced by NaCl, H_2_O_2_, polyethylene glycol (PEG) and exogenous ABA. MdBBX10 improves drought tolerance possibly through increasing ABA responsive genes including *RD29A* and *HY5*. Cold promotes the accumulation of phosphorylated CRY2 which completely interacts with COP1 to release HY5. The accumulated HY5 directly binds to the promoter of *BBX7* and *BBX8*, leading to enhance cold tolerance. Drought, PEG and ABA upregulate the expression of *CmBBX22*, which in turn increases the transcription of *HY5* and *ABI5*, improving drought tolerance. The expression of *MdBBX37* is rapidly increased in response to cold and ABA treatments. MdBBX37 binds to the promoter of *MdCBFs* to activate their transcription, as well as interacts with MdICE1 to enhance the transcriptional activity of MdICE1 on *MdCBF1*, resulting in promoting cold tolerance. Drought and cold stimulate the expression of *CmBBX24.* The accumulated CmBBX24 positively regulate genes related to compatible solutes and carbohydrate metabolism to strength tolerance to drought and cold stress, but negatively regulate GA biosynthesis to delay flowering. Heat elevates the transcript and protein level of BBX18 and BBX23 which reduce the protein accumulation of ELF3, releasing the PIF4-mediated hypocotyl growth in thermomorphogenesis. However, BBX18 downregulates the expression of *HSP70* and *HSP101,* leading to weaken thermotolerance. BBX21 negatively regulates dehydration response, while BBX29 augments drought tolerance in sugarcane. BBX24 and GbBBX25 are involved in strengthening salt tolerance

Light and phytohormone are critical issues to adjust plant growth and development. However, environmental cues such as drought, salinity, flooding, cold, high temperature, and other abiotic stresses also tightly control plant growth and development. Accumulated studies demonstrate that BBX proteins play critical role in abiotic stress response (Fig. 6). BBX24, named as salt tolerance protein (STO), which is identified as a protein conferring salt tolerance in yeast (Lippuner et al. 1996). Overexpression of *STO* enhances root growth tolerance to high salinity, implicating STO is involved in salt-stress response (Nagaoka and Takano 2003). BBX25 is known as salt tolerance homolog (STH) which plays additive role with BBX24 during deetiolation and shade avoidance response. Ectopic expression ginkgo (*Ginkgo biloba* L.) *BBX25* in populus enhances salt tolerance with greater sugar levels and higher peroxidase activity (Huang et al. 2021). BBX21/STH2 is also a homolog of STO and STH. Molecular lesions in *STH2* lead to reduce stomatal aperture and water loss under ABA and NaCl treatment, indicating *sth2* mutants are hypersensitive to ABA and salt stress. Besides, STH2 interacts with HY5 and ABI5 to interfere with their binding ability to the promoter of *ABI5,* contributing to decelerate ABA response. Therefore, STH2 negatively regulates ABA-mediated dehydration tolerance (Xu et al. 2014). In addition, ABA is also usually used to hasten flowering through enhancing the transcriptional activity or expression of CO and/or FT in response to drought stress (Riboni et al. 2016).

Transgenic plants overexpression *AtBBX29* in sugarcane (*Saccharum spp. hybrid*) increase proline accumulation and enzymatic antioxidant activity to survive during dehydration. Besides, transgenic plants maintain a higher relative water content and photosynthetic rate under drought stress. Thus, heterologous expression of *AtBBX29* in sugarcane enhances drought tolerance and delays senescence under water-deficit conditions (Mbambalala et al. 2021). CmBBX19 interacts with CmABF3 to repress ABA response, leading to reduce drought tolerance (Xu et al. 2020), while CmBBX22 enhances drought tolerance possibly via increasing the expression of *ABI3*, *ABI5* and *HY5* (Liu et al. 2019a, b). CmBBX24 delays flowering and improves tolerance to freezing and drought stress partially through negatively regulating GA biosynthesis and positively regulating genes related to compatible solutes and carbohydrate metabolism which are associated with abiotic stress (Yang et al. 2014). Exogenous ABA, NaCl, H_2_O_2_ and polyethylene glycol (PEG) significantly induce the expression of *Malus domestica BBX10 (MdBBX10)*. Ectopic expression *MdBBX10* in Arabidopsis markedly augments tolerance to drought and salt stress with higher germination ratio and longer root length. Besides, MdBBX10 enhances the ability of scavenging reactive oxygen species (ROS) under stress through improving the expression of ROS-related genes such as superoxide dismutase (*SOD*), ascorbateperoxidase (*APX*) and glutathione s-transferase (*GST*) (Liu et al. 2019a, b).

Mutations in *BBX7* and *BBX8* show freezing sensitivity, while overexpression of *BBX7* and *BBX8* enhance freezing tolerance, reflecting the positive role of BBX7 and BBX8 at cold temperature. Cold enhances the stability of blue light-induced phosphorylated CRY2 which competitively binds to COP1 to attenuate the COP1-HY5 interaction, allowing the accumulation of HY5 which directly binds to the promoter of *BBX7* and *BBX8* at cold stress. BBX7 and BBX8 positively control freezing tolerance by regulating genes related to the biosynthesis and metabolism of anthocyanins, flavonoids, and aromatic compounds which are independently of C-repeat-binding (CBF) factor pathway (Li et al. 2021b). Thus, BBX7 and BBX8 act downstream of CRY2-COP1-HY5 module to positively regulate blue light-dependent cold acclimation.

Warm temperature (for example 29℃) induces the transcript and protein level of BBX18 and BBX23, which interact with ELF3 to repress its protein accumulation. The decreased ELF3 relieves PIF4-mediated hypocotyl growth to promote thermomophogenesis. Thereby, BBX18 and BBX23 positively regulate thermomophogenic development in a ELF3 dependent manner (Ding et al. 2018). However, BBX18 is also documented to associate with PRR5 to alleviate the PRR5-mediated suppression of PIF4 activity which strengths the thermoresponsive hypocotyl growth in a ELF3 independent pathway (Hwang et al. 2021). Thus, plants employ BBX18 to adapt to elevated ambient temperature both in ELF3 dependent and independent pathway. However, in response to heat stress (for example 42℃), BBX18 limits the expression of heat stress related genes including *Hsp70*, *Hsp101* and *APX2*. Consistently, RNA interference-mediated under-expression of *BBX18* transgenic lines increase both basal and acquired thermotolerance with higher seed germination and survival rate, while overexpression of *BBX18* decrease tolerance to heat stress in transgenic plants. Collectively, BBX18 positively regulates thermomophogenesis but negatively modulates thermotolerance (Wang et al. 2013b, a).

## Other functions of the BBX proteins

In addition to the functions of BBX proteins in photomorphogenesis, flowering, shade avoidance, hormonal signaling pathway and stress response, increased studies reveal that they also play key role in circadian rhythm, senescence, and anthocyanin biosynthesis. PRR9, PRR7, and PRR5 interact with BBX19 to increase its binding ability to the promoter of morning-phased clock genes such as *CCA1*, *LHY1*, and *RVE8,* enhancing the repressive effect on the morning-phased clock genes. Thus, BBX19 acts as a crucial regulator to interact with PRR9/7/5 proteins to fine-tune the circadian rhythm (Yuan et al. 2021). Transgenic plants overexpression *Rosa hybrida BBX28* (*RhBBX28*) delay flower senescence and deduce H_2_O_2_ accumulation, while silencing *RhBBX28* has the opposite effects. The transcription level of *RhBBX28* is associated with H_2_O_2_ level which exhibits typical circadian rhythmicity in rose. RhBBX28 controls the expression of genes related to respiratory metabolism which play vital role in mitochondrial ROS homeostasis. In addition, RhPIF8 promotes the expression of *RhBBX28* to inhibit H_2_O_2_ levels in petals and thus delay flower senescence (Zhang et al. 2021).

Sixty-four apple BBX (*Malus domestica* Borkh) were identified by comprehensive bioinformatics analysis, many of them are identified to response to anthocyanin biosynthesis. Upon increased temperature and UV-B exposure, MdCOL11/MdBBX33, which acts downstream of MdHY5, directly binds to the promoter of *MdMYBA* to promote anthocyanin accumulation in apple peel. In addition, MdBBX1, MdBBX15, MdBBX17, MdBBX35, MdBBX51, and MdBBX54 activate the transcription of *MYB10* which is correlation with anthocyanin biosynthesis (Plunkett et al. 2019). MdBBX1 not only activates *MYB10* expression, but also strengths MYB10-activated *DFR* expression. MdBBX20 promotes anthocyanin biosynthesis through interacting with MdHY5 to enhance the transcription of *MdMYB1* (Fang et al. 2019). MdBBX23 directly binds to the promoter of *MdHY5* to activate its transcription, leading to the hypocotyl inhibition and anthocyanin accumulation. MdBBX37 interferes with the binding ability of MdMYB1 and MdMYB9 to their targets to attenuate anthocyanin accumulation via physical interactions. MdBBX37 directly binds to the promoter of *MdHY5* to suppress its expression, leading to hypocotyl elongation (An et al. 2020). Moreover, MdBBX37 not only binds to the promoter of *MdCBF1* and *MdCBF4* to activate their transcription, but also interacts with Malus domestica INDUCER OF CBF EXPRESSION 1 (MdICE1) to enhance the transcriptional activity of MdICE1 on *MdCBF1*, leading to promote cold tolerance. Malus domestica JASMONATE-ZIM-DOMAIN PROTEIN 1 (MdJAZ1) and MdJAZ2 interact with MdBBX37 to repress its transcriptional activity and interfere with the interaction between MdBBX37 and MdICE1, attenuating JA-mediated cold tolerance. In addition, MdMIEL1 acts as a RING type E3 ligase to target MdBBX37 for ubiquitination and degradation (An et al. 2021). PpBBX16 is identified as a positive regulator of anthocyanin biosynthesis. PpBBX16 interacts with PpHY5 to enhance the activation of *PpMYB10* and *PpCHS*, contributing to the accumulation of anthocyanin (Bai et al. 2019c). Similarly, PpBBX18 interacts with PpHY5 to activate *PpMYB10* expression and anthocyanin biosynthesis, whereas PpBBX21 disrupts the interaction between PpBBX18 and PpHY5 through directly association with them to reduce anthocyanin biosynthesis (Bai et al. 2019b).

## Concluding marks and future perspectives

BBX proteins play important role in a multitude of physiological process throughout the plant life cycle, including photomorphogenesis, flowering, anthocyanin biosynthesis, shade avoidance, phytohormone-mediated programs, biotic and abiotic stress and so on. In this review, we summarize the current existing studies of multi-layered roles of BBX proteins in diverse developmental processes, providing a comprehensive signal transduction network of BBX proteins-mediated plant growth and development. However, multiple remaining BBXs await investigation in detail, especially in crop plants.

Accumulated studies have identified a wide range of BBX proteins in various plant species such as rice, wheat, maize, soybean, cotton, potato, tomato, pear, rose, apple, bananas, chrysanthemum, grapevine, peanut, sweet cherry, strawberry, peach, raspgerry, and Moso bamboo. 8 of the 30 OsBBX proteins in rice are reported to play critical role in floral transition, however most of them are remained to be studied in detail. 10 MdBBX proteins have been identified and characterized in apple, while the remaining 54 MdBBX proteins need further investigation. 2 PpBBXs have been studied in pear, whereas the function of 35 PpBBX proteins remain unknown. 24 members of grapevine BBX (VvBBX) were identified, VvBBX22 is supposed to participate in multiple functions, including leaf senescence, abiotic stress responses, fruit development, and hormone response, however the role of the remaining VvBBX proteins need to be dissected (Crocco and Botto 2013). Since BBXs are evolutionarily conserved in various plant species. Functional analysis of BBXs in multiple plant species will help us better understand the role of the conserved and evolutionary BBX family, which will add new insights into crop breeding. Actually, some BBX proteins have shown great potentials in improving agronomic traits. Solanum lycopersicum BBX20 (SlBBX20) directly binds to the G-box motif of the promoter of *PHYTOENE SYNTHASE 1 (PSY1)* to activate its expression, contributing to the accumulation of carotenoid in tomato. Consistently, overexpression of *SlBBX20* leads to dark green fruits and leaves and higher levels of carotenoids, which is a new target for genetic improvement of the nutritional quality of tomato fruit (Xiong et al. 2019). In addition, SlBBX20 and SlBBX21 interact with SlHY5 to bind to the *SlHY5* promoter to activate its expression, whereas accumulated SlHY5 outcompetes SlBBX20 for binding to the *SlHY5* promoter to negatively regulate its own transcription under UV-B. Such autoregulatory negative feedback loop fine-tunes seedling establishment in response to UV-B light (Yang et al. 2022). Ectopic expression of *AtBBX21* in potato (*Solanum tuberosum*) promotes photosynthesis, anthocyanin accumulation and tuber yield which is more robust and shorter than wild-type plant. Heterologous expression of *AtBBX32* in soybean (*Glycine max*) results in increased duration of the pod and seed development and grain yield. Overexpression of *GmBBX52* and *GmBBX53* enhance yield and delay maturation similarity to what is observed in soybean plants overexpressing *AtBBX32* (Preuss et al. 2012). Transgenic rice with overexpression of *OsCOL9* enhance resistance to *Magnaporthe oryzae*, while knock out of *OsCOL9* with clustered regularly interspaced short palindromic repeats (CRISPR) are more susceptible to blast (Liu et al. 2016a, b). Considering the crucial roles of BBXs in the control of plant growth and development, and manipulation of BBXs with modern biotechnologies such as transgenic and CRISPR-mediated genome editing are available. It is possible to generate desirable agronomic traits in diverse crops by manipulating BBXs with advanced biotechnologies in future.

## Data Availability

Not applicable.

## Abbreviations

ABA: ABSCISIC ACID
ABF3: ABSCISIC ACID RESPONSIVE ELEMENTS-BINDING FACTOR 3
BBX: B-BOX DOMAIN PROTEIN
BEE1: BR-ENHANCED EXPRESSION 1
BEE2: BR-ENHANCED EXPRESSION 2
BEE3: BR-ENHANCED EXPRESSION 3
BR: BRASSINOSTEROID
BRZ: BRASSINAZOLE
BZR1: BRASSINAZOLE-RESISTANT 1
BZS1: Bzr1–1D SUPPRESSOR1-DOMINANT 1
CBF1: C-REPEAT/DRE BINDING FACTOR 1
CCA1: CIRCADIAN CLOCK ASSOCIATED 1
CCT: CONSTANSE, CONSTANSE-LIKE, AND TOC1
CO: CONSTANSE
COP1: CONSTITUTIVELY PHOTOMORPHOGENIC 1
CRY1: CRYPTOCHROME 1
CRY2: CRYPTOCHROME 2
DBB1a: DOUBLE B-BOX 1a
EHD1: EARLY HEADING DATE 1
ELF3: EARLY FLOWERING 3
FKF1: FLAVIN-BINDING KELCH REPEAT F-BOX 1
FLC: FLOWERING LOCUS C
FT: FLOWERING LOCUS T
GA: GIBBERELLIN ACID
GA3ox1: GIBBERELLIN3 BETA-HYDOXYGENASE 1
GA20ox1: GIBBERELLIN20-OXIDASE 1
GHD7: GRAIN NUMBER, PLANT HEIGHT AND HEADING DATE 7
GI: GIGANTEA
GLK1: GOLDEN2-LIKE 1
GUN1: GENOMES UNCOUPLED 1
HD1: HEADING DATE 1
HFR1: LONG HYPOCOTYL IN FAR-RED 1
HY5: ELONGATED HYPOCOTYL 5
ICE1: INDUCER OF CBF EXPRESSION 1
JAZ1: JASMONATE-ZIM-DOMAIN PROTEIN 1
JAZ2: JASMONATE-ZIM-DOMAIN PROTEIN 2
LHY1: LATE ELONGATED HYPOCOTYL 1
LKP2: LOV KELCH PROTEIN 2
NFYB2: NUCLEAR FACTOR Y, SUBUNIT B2
NF-YC3: NUCLEAR FACTOR Y, SUBUNIT C3
NYE1: NON-YELLOWING 1
NYE2: NON-YELLOWING 2
OBF4: OCTOPINE SYNTHASE-ELEMENT-BINDING FACTOR 4
PAR1: PHYTOCHROME RAPIDLY REGULATED 1
PIF1: PHYTOCHROME INTERACTING FACTOR 1
PIF3: PHYTOCHROME INTERACTING FACTOR 3
PIF4: PHYTOCHROME INTERACTING FACTOR 4
PIF5: PHYTOCHROME INTERACTING FACTOR 5
PIF7: PHYTOCHROME INTERACTING FACTOR 7
PIF8: PHYTOCHROME INTERACTING FACTOR 8
phyA: PHYTOCHROME A
phyB: PHYTOCHROME B
PIL1: PHYTOCHROME INTERACTING FACTOR 3-LIKE 1
PRR5: PSEUDO-RESPONSE REGULATOR 5
PRR7: PSEUDO-RESPONSE REGULATOR 7
PRR9: PSEUDO-RESPONSE REGULATOR 9
RBCC: RING, B-box, COILED-COIL
RFT1: RICE FLOWERING LOCUS T1
RVE8: REVEILLE 8
SA: SALICYLIC ACID
SAG29: SENESCENCE-ASSOCIATED GENE 29
SOC1: SUPPRESSOR OF OVEREXPRESSION OF CO 1
SPA: SUPPRESSOR OF PHYTOCHROME A
STH2: SALT TOLERANCE HOMOLOG2
STO: SALT TOLERANCE
SUR2: SUPERROOT 2
TGA4: TGACG MOTIF-BINDING FACTOR 4
TOC1: TIMING OF CAB EXPRESSION 1
ZTL: ZEITLUPE

## References

[CR1] An JP, Wang XF, Espley RV, Lin-Wang K, Bi SQ, You CX, Hao YJ (2020). An Apple B-Box Protein MdBBX37 Modulates Anthocyanin Biosynthesis and Hypocotyl Elongation Synergistically with MdMYBs and MdHY5. Plant Cell Physiol.

[CR2] An JP, Wang XF, Zhang XW, You CX, Hao YJ (2021). Apple B-box protein BBX37 regulates jasmonic acid mediated cold tolerance through the JAZ-BBX37-ICE1-CBF pathway and undergoes MIEL1-mediated ubiquitination and degradation. New Phytol.

[CR3] Bai B, Zhao J, Li YP, Zhang F, Zhou JJ, Chen F, Xie XZ (2016). OsBBX14 delays heading date by repressing florigen gene expression under long and short-day conditions in rice. Plant Sci.

[CR4] Bai MJ, Sun JJ, Liu JY, Ren HR, Wang K, Wang YL, Wang CQ, Dehesh K (2019). The B-box protein BBX19 suppresses seed germination via induction of ABI5. Plant J.

[CR5] Bai S, Tao R, Yin L, Ni J, Yang Q, Yan X, Yang F, Guo X, Li H, Teng Y (2019). Two B-box proteins, PpBBX18 and PpBBX21, antagonistically regulate anthocyanin biosynthesis via competitive association with Pyrus pyrifolia ELONGATED HYPOCOTYL 5 in the peel of pear fruit. Plant J.

[CR6] Bai S, Tao R, Tang Y, Yin L, Ma Y, Ni J, Yan X, Yang Q, Wu Z, Zeng Y, Teng Y (2019). BBX16, a B-box protein, positively regulates light-induced anthocyanin accumulation by activating MYB10 in red pear. Plant Biotechnol J.

[CR7] Bian Y, Chu L, Lin H, Qi Y, Fang Z, Xu DQ (2022). PIFs- and COP1-HY5-mediated temperature signaling in higher plants. Stress Biology.

[CR8] Bu X, Wang XJ, Yan JR, Zhang Y, Zhou SY, Sun X, Yang YX, Ahammed GJ, Liu YF, Qi MF, Wang F, Li TL (2021) Genome-Wide Characterization of B-Box Gene Family and Its Roles in Responses to Light Quality and Cold Stress in Tomato. Front Plant Sci 12:698525. 10.3389/fpls.2021.698525 10.3389/fpls.2021.698525 PMC8287887 34290726

[CR9] Bursch K, Toledo-Ortiz G, Pireyre M, Lohr M, Braatz C, Johansson H (2020). Identification of BBX proteins as rate-limiting cofactors of HY5. Nat Plants.

[CR10] Cao YP, Han YH, Meng DD, Li DH, Jiao CY, Jin Q, Lin Y, Cai YP (2017) B-BOX genes: genome-wide identification, evolution and their contribution to pollen growth in pear (Pyrus bretschneideri Rehd.). Bmc Plant Biol 17:156. 10.1186/s12870-017-1105-4 10.1186/s12870-017-1105-4 PMC5606111 28927374

[CR11] Cao J, Liang Y, Yan T, Wang X, Zhou H, Chen C, Zhang Y, Zhang B, Zhang S, Liao J, Cheng S, Chu J, Huang X, Xu D, Li J, Deng XW, Lin F (2022). The photomorphogenic repressors BBX28 and BBX29 integrate light and brassinosteroid signaling to inhibit seedling development in Arabidopsis. Plant Cell.

[CR12] Casal JJ (2013). Photoreceptor Signaling Networks in Plant Responses to Shade. Annu Rev Plant Biol.

[CR13] Chaurasia AK, Patil HB, Azeez A, Subramaniam VR, Krishna B, Sane AP, Sane PV (2016). Molecular characterization of CONSTANS-Like (COL) genes in banana (Musa acuminata L. AAA Group, cv. Grand Nain). Physiol Mol Biol Pla.

[CR14] Chen SH, Jiang WQ, Yin JL, Wang SP, Fang ZW, Ma DF, Gao DR (2021). Genome-wide mining of wheat B-BOX zinc finger (BBX) gene family provides new insights into light stress responses. Crop Pasture Sci.

[CR15] Cheng XF, Wang ZY (2005). Overexpression of COL9, a CONSTANS-LIKE gene, delays flowering by reducing expression of CO and FT in Arabidopsis thaliana. Plant J.

[CR16] Cheng MC, Kathare PK, Paik I, Huq E (2021). Phytochrome Signaling Networks. Annu Rev Plant Biol.

[CR17] Chu ZN, Wang X, Li Y, Yu HY, Li JH, Lu YG, Li HX, Ouyang B (2016). Genomic Organization, Phylogenetic and Expression Analysis of the B-BOX Gene Family in Tomato. Front Plant Sci.

[CR18] Crocco CD, Botto JF (2013). BBX proteins in green plants: Insights into their evolution, structure, feature and functional diversification. Gene.

[CR19] Crocco CD, Holm M, Yanovsky MJ, Botto JF (2011). Function of B-BOX under shade. Plant Signal Behav.

[CR20] Crocco CD, Locascio A, Escudero CM, Alabadi D, Blazquez MA, Botto JF (2015). The transcriptional regulator BBX24 impairs DELLA activity to promote shade avoidance in Arabidopsis thaliana. Nat Commun.

[CR21] Datta S, Hettiarachchi GHCM, Deng XW, Holm M (2006). Arabidopsis CONSTANS-LIKE3 is a positive regulator of red light signaling and root growth. Plant Cell.

[CR22] Datta S, Hettiarachchi C, Johansson H, Holm M (2007). SALT TOLERANCE HOMOLOG2, a B-Box protein in Arabidopsis that activates transcription and positively regulates light-mediated development. Plant Cell.

[CR23] Ding L, Wang S, Song ZT, Jiang YP, Han JJ, Lu SJ, Li L, Liu JX (2018). Two B-Box Domain Proteins, BBX18 and BBX23, Interact with ELF3 and Regulate Thermomorphogenesis in Arabidopsis. Cell Rep.

[CR24] Fan XY, Sun Y, Cao DM, Bai MY, Luo XM, Yang HJ, Wei CQ, Zhu SW, Sun Y, Chong K, Wang ZY (2012). BZS1, a B-box protein, promotes photomorphogenesis downstream of both brassinosteroid and light signaling pathways. Mol Plant.

[CR25] Fan C, Hu R, Zhang X, Wang X, Zhang W, Zhang Q, Ma J, Fu YF (2014). Conserved CO-FT regulons contribute to the photoperiod flowering control in soybean. BMC Plant Biol.

[CR26] Fang H, Dong Y, Yue X, Hu J, Jiang S, Xu H, Wang Y, Su M, Zhang J, Zhang Z, Wang N, Chen X (2019). The B-box zinc finger protein MdBBX20 integrates anthocyanin accumulation in response to ultraviolet radiation and low temperature. Plant Cell Environ.

[CR27] Feng Z, Li MY, Li Y, Yang X, Wei HL, Fu XK, Ma L, Lu JH, Wang HT, Yu SX (2021). Comprehensive identification and expression analysis of B-Box genes in cotton. BMC Genomics.

[CR28] Gangappa SN, Botto JF (2014). The BBX family of plant transcription factors. Trends Plant Sci.

[CR29] Gangappa SN, Holm M, Botto JF (2013a) Molecular interactions of BBX24 and BBX25 with HYH, HY5 HOMOLOG, to modulate Arabidopsis seedling development. Plant Signal Behav 8:e25208. 10.4161/psb.25208 10.4161/psb.25208 PMC3999086 23733077

[CR30] Gangappa SN, Crocco CD, Johansson H, Datta S, Hettiarachchi C, Holm M, Botto JF (2013). The Arabidopsis B-BOX protein BBX25 interacts with HY5, negatively regulating BBX22 expression to suppress seedling photomorphogenesis. Plant Cell.

[CR31] Gnesutta N, Kumimoto RW, Swain S, Chiara M, Siriwardana C, Horner DS, Holt BF, Mantovani R (2017). CONSTANS Imparts DNA Sequence Specificity to the Histone Fold NF-YB/NF-YC Dimer. Plant Cell.

[CR32] Graeff M, Straub D, Eguen T, Dolde U, Rodrigues V, Brandt R, Wenkel S (2016) MicroProtein-Mediated Recruitment of CONSTANS into a TOPLESS Trimeric Complex Represses Flowering in Arabidopsis. PLoS Genet 12:e1005959. 10.1371/journal.pgen.1005959 10.1371/journal.pgen.1005959 PMC4807768 27015278

[CR33] Hassidim M, Harir Y, Yakir E, Kron I, Green RM (2009). Over-expression of CONSTANS-LIKE 5 can induce flowering in short-day grown Arabidopsis. Planta.

[CR34] Heng Y, Jiang Y, Zhao X, Zhou H, Wang X, Deng XW, Xu D (2019). BBX4, a phyB-interacting and modulated regulator, directly interacts with PIF3 to fine tune red light-mediated photomorphogenesis. Proc Natl Acad Sci U S A.

[CR35] Heng Y, Lin F, Jiang Y, Ding M, Yan T, Lan H, Zhou H, Zhao X, Xu D, Deng XW (2019). B-Box Containing Proteins BBX30 and BBX31, Acting Downstream of HY5, Negatively Regulate Photomorphogenesis in Arabidopsis. Plant Physiol.

[CR36] Holm M, Deng XW (1999). Structural organization and interactions of COP1, a light-regulated developmental switch. Plant Mol Biol.

[CR37] Holm M, Hardtke CS, Gaudet R, Deng XW (2001). Identification of a structural motif that confers specific interaction with the WD40 repeat domain of Arabidopsis COP1. EMBO J.

[CR38] Holtan HE, Bandong S, Marion CM, Adam L, Tiwari S, Shen Y, Maloof JN, Maszle DR, Ohto MA, Preuss S, Meister R, Petracek M, Repetti PP, Reuber TL, Ratcliffe OJ, Khanna R (2011). BBX32, an Arabidopsis B-Box Protein, Functions in Light Signaling by Suppressing HY5-Regulated Gene Expression and Interacting with STH2/BBX21. Plant Physiol.

[CR39] Huang JY, Zhao XB, Weng XY, Wang L, Xie WB (2012) The Rice B-Box Zinc Finger Gene Family: Genomic Identification, Characterization, Expression Profiling and Diurnal Analysis. PLoS ONE 7:e48242. 10.1371/journal.pone.0048242 10.1371/journal.pone.0048242 PMC3485221 23118960

[CR40] Huang X, Zhang Q, Jiang YP, Yang CW, Wang QY, Li L (2018) Shade-induced nuclear localization of PIF7 is regulated by phosphorylation and 14–3–3 proteins in Arabidopsis. Elife 7:e31636. 10.7554/elife.31636 10.7554/eLife.31636 PMC6037483 29926790

[CR41] Huang S, Chen C, Xu M, Wang G, Xu LA, Wu Y (2021). Overexpression of Ginkgo BBX25 enhances salt tolerance in Transgenic Populus. Plant Physiol Biochem.

[CR42] Hwang G, Park J, Kim S, Park J, Seo D, Oh E (2021) Overexpression of BBX18 Promotes Thermomorphogenesis Through the PRR5-PIF4 Pathway. Front Plant Sci 12:782352. 10.3389/fpls.2021.782352 10.3389/fpls.2021.782352 PMC8651621 34899810

[CR43] Jiang L, Wang Y, Li QF, Bjorn LO, He JX, Li SS (2012). Arabidopsis STO/BBX24 negatively regulates UV-B signaling by interacting with COP1 and repressing HY5 transcriptional activity. Cell Res.

[CR44] Jiao Y, Lau OS, Deng XW (2007). Light-regulated transcriptional networks in higher plants. Nat Rev Genet.

[CR45] Jin HQ, Xing MG, Cai CM, Li S (2020). B-box Proteins in Arachis duranensis: Genome-Wide Characterization and Expression Profiles Analysis. Agronomy-Basel.

[CR46] Job N, Datta S (2021). PIF3/HY5 module regulates BBX11 to suppress protochlorophyllide levels in dark and promote photomorphogenesis in light. New Phytol.

[CR47] Kami C, Lorrain S, Hornitschek P, Fankhauser C (2010). Light-regulated plant growth and development. Curr Top Dev Biol.

[CR48] Khanna R, Kronmiller B, Maszle DR, Coupland G, Holm M, Mizuno T, Wu SH (2009). The Arabidopsis B-box zinc finger family. Plant Cell.

[CR49] Kim SK, Yun CH, Lee JH, Jang YH, Park HY, Kim JK (2008). OsCO3, a CONSTANS-LIKE gene, controls flowering by negatively regulating the expression of FT-like genes under SD conditions in rice. Planta.

[CR50] Lau OS, Deng XW (2012). The photomorphogenic repressors COP1 and DET1: 20 years later. Trends Plant Sci.

[CR51] Lee YS, Jeong DH, Lee DY, Yi J, Ryu CH, Kim SL, Jeong HJ, Choi SC, Jin P, Yang J, Cho LH, Choi H, An G (2010) OsCOL4 is a constitutive flowering repressor upstream of Ehd1 and downstream of OsphyB. Plant J 63:18–30. 10.1111/j.1365-313x.2010.04226.x 10.1111/j.1365-313X.2010.04226.x 20409004

[CR52] Leivar P, Monte E (2014). PIFs: Systems Integrators in Plant Development. Plant Cell.

[CR53] Leivar P, Monte E, Al-Sady B, Carle C, Storer A, Alonso JM, Ecker JR, Quail PH (2008). The Arabidopsis phytochrome-interacting factor PIF7, together with PIF3 and PIF4, regulates responses to prolonged red light by modulating phyB levels. Plant Cell.

[CR54] Li L, Ljung K, Breton G, Schmitz RJ, Pruneda-Paz J, Cowing-Zitron C, Cole BJ, Ivans LJ, Pedmale UV, Jung HS, Ecker JR, Kay SA, Chory J (2012). Linking photoreceptor excitation to changes in plant architecture. Gene Dev.

[CR55] Li F, Su JJ, Wang DH, Bai SN, Clarke AK, Holm M (2014) The B-Box Family Gene STO (BBX24) in Arabidopsis thaliana Regulates Flowering Time in Different Pathways. PLoS ONE 9:e87544. 10.1371/journal.pone.0087544 10.1371/journal.pone.0087544 PMC3911981 24498334

[CR56] Li TT, Li BB, Wang LZ, Xie ZH, Wang XT, Zou LJ, Zhang DW, Lin HH (2021). Phytochrome-interacting factor 4 (PIF4) inhibits expression of SHORT HYPOCOTYL 2 (SHY2) to promote hypocotyl growth during shade avoidance in Arabidopsis. Biochem Bioph Res Co.

[CR57] Li YP, Shi YT, Li MZ, Fu DY, Wu SF, Li JG, Gong ZZ, Liu HT, Yang SH (2021). The CRY2-COP1-HY5-BBX7/8 module regulates blue light-dependent cold acclimation in Arabidopsis. Plant Cell.

[CR58] Lin F, Jiang Y, Li J, Yan T, Fan L, Liang J, Chen ZJ, Xu D, Deng XW (2018). B-BOX DOMAIN PROTEIN28 Negatively Regulates Photomorphogenesis by Repressing the Activity of Transcription Factor HY5 and Undergoes COP1-Mediated Degradation. Plant Cell.

[CR59] Lin F, Cao J, Yuan JL, Liang YX, Li J (2021). Integration of Light and Brassinosteroid Signaling during Seedling Establishment. Int J Mol Sci.

[CR60] Lippuner V, Cyert MS, Gasser CS (1996). Two classes of plant cDNA clones differentially complement yeast calcineurin mutants and increase salt tolerance of wild-type yeast. J Biol Chem.

[CR61] Liu H, Dong S, Sun D, Liu W, Gu F, Liu Y, Guo T, Wang H, Wang J, Chen Z (2016a) CONSTANS-Like 9 (OsCOL9) Interacts with Receptor for Activated C-Kinase 1(OsRACK1) to Regulate Blast Resistance through Salicylic Acid and Ethylene Signaling Pathways. PLoS ONE 11:e0166249. 10.1371/journal.pone.0166249 10.1371/journal.pone.0166249 PMC5102437 27829023

[CR62] Liu H, Gu FW, Dong SY, Liu W, Wang H, Chen ZQ, Wang JF (2016). CONSTANS-like 9 (COL9) delays the flowering time in Oryza sativa by repressing the Ehd1 pathway. Biochem Bioph Res Co.

[CR63] Liu X, Li R, Dai Y, Chen X, Wang X (2018). Genome-wide identification and expression analysis of the B-box gene family in the Apple (*Malus domestica* Borkh.) genome. Mol Genet Genomics.

[CR64] Liu X, Li R, Dai Y, Yuan L, Sun Q, Zhang S, Wang X (2019). A B-box zinc finger protein, MdBBX10, enhanced salt and drought stresses tolerance in Arabidopsis. Plant Mol Biol.

[CR65] Liu YN, Chen H, Ping Q, Zhang ZX, Guan ZY, Fang WM, Chen SM, Chen FD, Jiang JF, Zhang F (2019). The heterologous expression of CmBBX22 delays leaf senescence and improves drought tolerance in Arabidopsis. Plant Cell Rep.

[CR66] Liu Y, Lin G, Yin CM, Fang YD (2020). B-box transcription factor 28 regulates flowering by interacting with constans. Sci Rep-Uk.

[CR67] Lorrain S, Allen T, Duek PD, Whitelam GC, Fankhauser C (2008). Phytochrome-mediated inhibition of shade avoidance involves degradation of growth-promoting bHLH transcription factors. Plant J.

[CR68] Ma R, Chen J, Huang B, Huang Z, Zhang Z (2021). The BBX gene family in Moso bamboo (Phyllostachys edulis): identification, characterization and expression profiles. BMC Genomics.

[CR69] Mbambalala N, Panda SK, van der Vyver C (2021). Overexpression ofAtBBX29 improves drought tolerance by maintaining photosynth-esis and enhancing the antioxidant and osmolyte capacity of sugar-cane plants. Plant Mol Biol Report.

[CR70] Nagaoka S, Takano T (2003). Salt tolerance-related protein STO binds to a Myb transcription factor homologue and confers salt tolerance in Arabidopsis. J Exp Bot.

[CR71] Ordonez-Herrera N, Trimborn L, Menje M, Henschel M, Robers L, Kaufholdt D, Hansch R, Adrian J, Ponnu J, Hoecker U (2018). The Transcription Factor COL12 Is a Substrate of the COP1/SPA E3 Ligase and Regulates Flowering Time and Plant Architecture. Plant Physiol.

[CR72] Pacin M, Legris M, Casal JJ (2013). COP1 re-accumulates in the nucleus under shade. Plant J.

[CR73] Pierik R, de Wit M (2014). Shade avoidance: phytochrome signalling and other aboveground neighbour detection cues. J Exp Bot.

[CR74] Ping Q, Cheng PL, Huang F, Ren LP, Cheng H, Guan ZY, Fang WM, Chen SM, Chen FD, Jiang JF (2019). The heterologous expression in Arabidopsis thaliana of a chrysanthemum gene encoding the BBX family transcription factor CmBBX13 delays flowering. Plant Physiol Bioch.

[CR75] Plunkett BJ, Henry-Kirk R, Friend A, Diack R, Helbig S, Mouhu K, Tomes S, Dare AP, Espley RV, Putterill J, Allan AC (2019). Apple B-box factors regulate light-responsive anthocyanin biosynthesis genes. Sci Rep.

[CR76] Preuss SB, Meister R, Xu Q, Urwin CP, Tripodi FA, Screen SE, Anil VS, Zhu S, Morrell JA, Liu G, Ratcliffe OJ, Reuber TL, Khanna R, Goldman BS, Bell E, Ziegler TE, McClerren AL, Ruff TG, Petracek ME (2012) Expression of the Arabidopsis thaliana BBX32 gene in soybean increases grain yield. PLoS ONE 7:e30717. 10.1371/journal.pone.0030717 10.1371/journal.pone.0030717 PMC3281879 22363475

[CR77] Ravindran N, Ramachandran H, Job N, Yadav A, Vaishak KP, Datta S (2021). B-box protein BBX32 integrates light and brassinosteroid signals to inhibit cotyledon opening. Plant Physiol.

[CR78] Reymond A, Meroni G, Fantozzi A, Merla G, Cairo S, Luzi L, Riganelli D, Zanaria E, Messali S, Cainarca S, Guffanti A, Minucci S, Pelicci PG, Ballabio A (2001). The tripartite motif family identifies cell compartments. EMBO J.

[CR79] Riboni M, Test AR, Galbiati M, Tonelli C, Conti L (2016). ABA-dependent control of GIGANTEA signalling enables drought escape via up-regulation of FLOWERING LOCUS T in Arabidopsis thaliana. J Exp Bot.

[CR80] Sarmiento F (2013) The BBX subfamily IV: additional cogs and sprockets to fine-tune light-dependent development. Plant Signal Behav 8:e23831. 10.4161/psb.23831 10.4161/psb.23831 PMC7030190 23425851

[CR81] Shalmani A, Fan S, Jia P, Li GF, Muhammad I, Li YM, Sharif R, Dong F, Zuo XY, Li K, Chen KM, Han MY (2018). Genome Identification of B-BOX Gene Family Members in Seven Rosaceae Species and Their Expression Analysis in Response to Flower Induction in Malus domestica. Molecules.

[CR82] Shalmani A, Jing XQ, Shi Y, Muhammad I, Zhou MR, Wei XY, Chen QQ, Li WQ, Liu WT, Chen KM (2019). Characterization of B-BOX gene family and their expression profiles under hormonal, abiotic and metal stresses in Poaceae plants. BMC Genomics.

[CR83] Sheng PK, Wu FQ, Tan JJ, Zhang H, Ma WW, Chen LP, Wang JC, Wang J, Zhu SS, Guo XP, Wang JL, Zhang X, Cheng ZJ, Bao YQ, Wu CY, Liu XM, Wan JM (2016). A CONSTANS-like transcriptional activator, OsCOL13, functions as a negative regulator of flowering downstream of OsphyB and upstream of Ehd1 in rice. Plant Mol Biol.

[CR84] Simon R, Igeno MI, Coupland G (1996). Activation of floral meristem identity genes in Arabidopsis. Nature.

[CR85] Song YH, Song NY, Shin SY, Kim HJ, Yun DJ, Lim CO, Lee SY, Kang KY, Hong JC (2008). Isolation of CONSTANS as a TGA4/OBF4 interacting protein. Mol Cells.

[CR86] Song Z, Bian Y, Liu J, Sun Y, Xu D (2020). B-box proteins: Pivotal players in light-mediated development in plants. J Integr Plant Biol.

[CR87] Song Z, Heng Y, Bian Y, Xiao Y, Liu J, Zhao X, Jiang Y, Deng XW, Xu D (2021). BBX11 promotes red light-mediated photomorphogenic development by modulating phyB-PIF4 signaling. Abiotech.

[CR88] Song Z, Yan T, Liu J, Bian Y, Heng Y, Lin F, Jiang Y, Deng XW, Xu D (2020). BBX28/BBX29, HY5 and BBX30/31 form a feedback loop to fine-tune photomorphogenic development. Plant J.

[CR89] Steinbach Y (2019). The Arabidopsis thaliana CONSTANS-LIKE 4 (COL4) - A Modulator of Flowering Time. Front Plant Sci.

[CR90] Talar U, Kielbowicz-Matuk A (2021). Beyond Arabidopsis: BBX Regulators in Crop Plants. Int J Mol Sci.

[CR91] Talar U, Kielbowicz-Matuk A, Czarnecka J, Rorat T (2017) Genome-wide survey of B-box proteins in potato (Solanum tuberosum)-Identification, characterization and expression patterns during diurnal cycle, etiolation and de-etiolation. PLoS ONE 12:e0177471. 10.1371/journal.pone.0177471 10.1371/journal.pone.0177471 PMC5446133 28552939

[CR92] Tan JJ, Jin MN, Wang JC, Wu FQ, Sheng PK, Cheng ZJ, Wang JL, Zheng XM, Chen LP, Wang M, Zhu SS, Guo XP, Zhang X, Liu XM, Wang CM, Wang HY, Wu CY, Wan JM (2016). OsCOL10, a CONSTANS-Like Gene, Functions as a Flowering Time Repressor Downstream of Ghd7 in Rice. Plant Cell Physiol.

[CR93] Tan JJ, Wu FQ, Wan JM (2017) Flowering time regulation by the CONSTANS-Like gene OsCOL10. Plant Signal Behav 12:e1267893. 10.1080/15592324.2016.1267893 10.1080/15592324.2016.1267893 PMC5289515 28095114

[CR94] Tiwari SB, Shen Y, Chang HC, Hou Y, Harris A, Ma SF, McPartland M, Hymus GJ, Adam L, Marion C, Belachew A, Repetti PP, Reuber TL, Ratcliffe OJ (2010). The flowering time regulator CONSTANS is recruited to the FLOWERING LOCUS T promoter via a unique cis-element. New Phytol.

[CR95] Tripathi P, Carvallo M, Hamilton EE, Preuss S, Kay SA (2017). Arabidopsis B-BOX32 interacts with CONSTANS-LIKE3 to regulate flowering. Proc Natl Acad Sci U S A.

[CR96] Vaishak KP, Yadukrishnan P, Bakshi S, Kushwaha AK, Ramachandran H, Job N, Babu D, Datta S (2019). The B-box bridge between light and hormones in plants. J Photoch Photobio B.

[CR97] Veciana N, Martin G, Leivar P, Monte E (2022). BBX16 mediates the repression of seedling photomorphogenesis downstream of the GUN1/GLK1 module during retrograde signalling. New Phytol.

[CR98] Wang QM, Zeng JX, Deng KQ, Tu XJ, Zhao XY, Tang DY, Liu XM (2011). DBB1a, involved in gibberellin homeostasis, functions as a negative regulator of blue light-mediated hypocotyl elongation in Arabidopsis. Planta.

[CR99] Wang HG, Zhang ZL, Li HY, Zhao XY, Liu XM, Ortiz M, Lin CT, Liu B (2013). CONSTANS-LIKE 7 regulates branching and shade avoidance response in Arabidopsis. J Exp Bot.

[CR100] Wang QM, Tu XJ, Zhang JH, Chen XB, Rao LQ (2013). Heat stress-induced BBX18 negatively regulates the thermotolerance in Arabidopsis. Mol Biol Rep.

[CR101] Wang CQ, Guthrie C, Sarmast MK, Dehesh K (2014). BBX19 Interacts with CONSTANS to Repress FLOWERING LOCUS T Transcription, Defining a Flowering Time Checkpoint in Arabidopsis. Plant Cell.

[CR102] Wang CQ, Sarmast MK, Jiang J, Dehesh K (2015). The Transcriptional Regulator BBX19 Promotes Hypocotyl Growth by Facilitating COP1-Mediated EARLY FLOWERING3 Degradation in Arabidopsis. Plant Cell.

[CR103] Wang LJ, Sun J, Ren LP, Zhou M, Han XY, Ding L, Zhang F, Guan ZY, Fang WM, Chen SM, Chen FD, Jiang JF (2020). CmBBX8 accelerates flowering by targeting CmFTL1 directly in summer chrysanthemum. Plant Biotechnol J.

[CR104] Wang YY, Zhai ZF, Sun YT, Feng C, Peng X, Zhang X, Xiao YQ, Zhou X, Wang WL, Jiao JL, Li TH (2021). Genome-Wide Identification of the B-BOX Genes that Respond to Multiple Ripening Related Signals in Sweet Cherry Fruit. Int J Mol Sci.

[CR105] Wei HR, Wang PP, Chen JQ, Li CJ, Wang YZ, Yuan YB, Fang JG, Leng XP (2020). Genome-wide identification and analysis of B-BOX gene family in grapevine reveal its potential functions in berry development. Bmc Plant Biol.

[CR106] Wu WX, Zheng XM, Chen DB, Zhang YX, Ma WW, Zhang H, Sun LP, Yang ZF, Zhao CD, Zhan XD, Shen XH, Yu P, Fu YP, Zhu SS, Cao LY, Cheng SH (2017). OsCOL16, encoding a CONSTANS-like protein, represses flowering by up regulating Ghd7 expression in rice. Plant Sci.

[CR107] Wu WX, Zhang YX, Zhang M, Zhan XD, Shen XH, Yu P, Chen DB, Liu QN, Sinumporn S, Hussain K, Cheng SH, Cao LY (2018). The rice CONSTANS-like protein OsCOL15 suppresses flowering by promoting Ghd7 and repressing RID1. Biochem Bioph Res Co.

[CR108] Xiong C, Luo D, Lin A, Zhang C, Shan L, He P, Li B, Zhang Q, Hua B, Yuan Z, Li H, Zhang J, Yang C, Lu Y, Ye Z, Wang T (2019). A tomato B-box protein SlBBX20 modulates carotenoid biosynthesis by directly activating PHYTOENE SYNTHASE 1, and is targeted for 26S proteasome-mediated degradation. New Phytol.

[CR109] Xu D (2020). COP1 and BBXs-HY5-mediated light signal transduction in plants. New Phytol.

[CR110] Xu D, Li J, Gangappa SN, Hettiarachchi C, Lin F, Andersson MX, Jiang Y, Deng XW, Holm M (2014) Convergence of Light and ABA signaling on the ABI5 promoter. PLoS Genet 10:e1004197. 10.1371/journal.pgen.1004197 10.1371/journal.pgen.1004197 PMC3937224 24586210

[CR111] Xu X, Paik I, Zhu L, Huq E (2015). Illuminating Progress in Phytochrome-Mediated Light Signaling Pathways. Trends Plant Sci.

[CR112] Xu D, Jiang Y, Li J, Lin F, Holm M, Deng XW (2016). BBX21, an Arabidopsis B-box protein, directly activates HY5 and is targeted by COP1 for 26S proteasome-mediated degradation. Proc Natl Acad Sci U S A.

[CR113] Xu D, Jiang Y, Li J, Holm M, Deng XW (2018). The B-Box Domain Protein BBX21 Promotes Photomorphogenesis. Plant Physiol.

[CR114] Xu YJ, Zhao X, Aiwaili P, Mu XY, Zhao M, Zhao J, Cheng LN, Ma C, Gao JP, Hong B (2020). A zinc finger protein BBX19 interacts with ABF3 to affect drought tolerance negatively in chrysanthemum. Plant J.

[CR115] Xu X, Li W, Yang S, Zhu X, Sun H, Li F, Lu X, Cui J (2022) Identification, evolution, expression and protein interaction analysis of genes encoding B-box zinc-finger proteins in maize. J Integr Agric. 10.1016/j.jia.2022.08.091

[CR116] Yadav A, Ravindran N, Singh D, Rahul PV, Datta S (2020). Role of Arabidopsis BBX proteins in light signaling. J Plant Biochem Biot.

[CR117] Yang Y, Ma C, Xu Y, Wei Q, Imtiaz M, Lan H, Gao S, Cheng L, Wang M, Fei Z, Hong B, Gao J (2014). A Zinc Finger Protein Regulates Flowering Time and Abiotic Stress Tolerance in Chrysanthemum by Modulating Gibberellin Biosynthesis. Plant Cell.

[CR118] Yang C, Xie F, Jiang Y, Li Z, Huang X, Li L (2018) Phytochrome A Negatively Regulates the Shade Avoidance Response by Increasing Auxin/Indole Acidic Acid Protein Stability. Dev Cell 44:29–41 e24. 10.1016/j.devcel.2017.11.017 10.1016/j.devcel.2017.11.017 29275991

[CR119] Yang G, Zhang C, Dong H, Liu X, Guo H, Tong B, Fang F, Zhao Y, Yu Y, Liu Y, Lin L, Yin R (2022). Activation and negative feedback regulation of SlHY5 transcription by the SlBBX20/21-SlHY5 transcription factor module in UV-B signaling. Plant Cell.

[CR120] Yano M, Katayose Y, Ashikari M, Yamanouchi U, Monna L, Fuse T, Baba T, Yamamoto K, Umehara Y, Nagamura Y, Sasaki T (2000). Hd1, a major photoperiod sensitivity quantitative trait locus in rice, is closely related to the arabidopsis flowering time gene CONSTANS. Plant Cell.

[CR121] Yuan L, Yu YJ, Liu MM, Song Y, Li HM, Sun JQ, Wang Q, Xie QG, Wang L, Xu XD (2021). BBX19 fine-tunes the circadian rhythm by interacting with PSEUDO-RESPONSE REGULATOR proteins to facilitate their repressive effect on morning-phased clock genes. Plant Cell.

[CR122] Zhang ZL, Ji RH, Li HY, Zhao T, Liu J, Lin CT, Liu B (2014). CONSTANS-LIKE 7 (COL7) Is Involved in Phytochrome B (phyB)-Mediated Light-Quality Regulation of Auxin Homeostasis. Mol Plant.

[CR123] Zhang X, Huai J, Shang F, Xu G, Tang W, Jing Y, Lin R (2017). A PIF1/PIF3-HY5-BBX23 Transcription Factor Cascade Affects Photomorphogenesis. Plant Physiol.

[CR124] Zhang Y, Wu ZC, Feng M, Chen JW, Qin MZ, Wang WR, Bao Y, Xu Q, Ye Y, Ma C, Jiang CZ, Gan SS, Zhou HG, Cai YM, Hong B, Gao JP, Ma N (2021). The circadian-controlled PIF8-BBX28 module regulates petal senescence in rose flowers by governing mitochondrial ROS homeostasis at night. Plant Cell.

[CR125] Zhao XH, Heng YQ, Wang XC, Deng XW, Xu DQ (2020) A Positive Feedback Loop of BBX11-BBX21-HY5 Promotes Photomorphogenic Development in Arabidopsis. Plant Commun 1:100045. 10.1016/j.xplc.2020.100045 10.1016/j.xplc.2020.100045 PMC7747993 33367254

